# Structural Maintenance of Chromosomes (SMC) Proteins Promote Homolog-Independent Recombination Repair in Meiosis Crucial for Germ Cell Genomic Stability

**DOI:** 10.1371/journal.pgen.1001028

**Published:** 2010-07-22

**Authors:** Jeremy S. Bickel, Liting Chen, Jin Hayward, Szu Ling Yeap, Ashley E. Alkers, Raymond C. Chan

**Affiliations:** 1 Department of Human Genetics, University of Michigan Medical School, Ann Arbor, Michigan, United States of America; 2 Department of Internal Medicine, Division of Molecular Medicine and Genetics, University of Michigan Medical School, Ann Arbor, Michigan, United States of America; Stowers Institute for Medical Research, United States of America

## Abstract

In meiosis, programmed DNA breaks repaired by homologous recombination (HR) can be processed into inter-homolog crossovers that promote the accurate segregation of chromosomes. In general, more programmed DNA double-strand breaks (DSBs) are formed than the number of inter-homolog crossovers, and the excess DSBs must be repaired to maintain genomic stability. Sister-chromatid (inter-sister) recombination is postulated to be important for the completion of meiotic DSB repair. However, this hypothesis is difficult to test because of limited experimental means to disrupt inter-sister and not inter-homolog HR in meiosis. We find that the conserved Structural Maintenance of Chromosomes (SMC) 5 and 6 proteins in *Caenorhabditis elegans* are required for the successful completion of meiotic homologous recombination repair, yet they appeared to be dispensable for accurate chromosome segregation in meiosis. Mutations in the *smc-5* and *smc-6* genes induced chromosome fragments and dismorphology. Chromosome fragments associated with HR defects have only been reported in mutants, which have disrupted inter-homolog crossover. Surprisingly, the *smc-5* and *smc-6* mutations did not disrupt the formation of chiasmata, the cytologically visible linkages between homologous chromosomes formed from meiotic inter-homolog crossovers. The mutant fragmentation defect appeared to be preferentially enhanced by the disruptions of inter-homolog recombination but not by the disruptions of inter-sister recombination. Based on these findings, we propose that the *C. elegans* SMC-5/6 proteins are required in meiosis for the processing of homolog-independent, presumably sister-chromatid-mediated, recombination repair. Together, these results demonstrate that the successful completion of homolog-independent recombination is crucial for germ cell genomic stability.

## Author Summary

Sperm and oocytes are essential for the faithful transmission of genetic information during sexual reproduction. As germ cells mature into sperm and oocytes, DNA double-strand breaks (DSBs) are deliberately created on each chromosome and a subset of DSBs is repaired to form meiotic crossovers between homologous chromosomes. Because germ cells must undergo this programmed process of deliberate DNA damage and repair, identifying repair factors active in germ cells and determining the requirement of their functions in meiotic DSB repair are important first steps in understanding infertility and developmental disorders caused by defective sperm and oocytes. In this manuscript, we find that the evolutionarily conserved SMC-5 and SMC-6 proteins fulfill a critical role in preserving genomic stability in germ cells in *C. elegans*. Our findings further describe the genetic mechanisms by which the *C. elegans* SMC-5/6 proteins function in meiotic DSB repair. These data reveal that inter-sister homologous recombination, a repair mechanism thought to function as a back-up repair method in meiosis, serves a more significant role in normal meiosis than was previously appreciated.

## Introduction

Homologous recombination (HR) utilizes an undamaged homologous DNA template to repair DNA double-strand breaks (DSBs). The use of template-mediated repair minimizes the likelihood of DNA sequence alterations arising during the repair process. For mitotic cells following DNA replication, the sister chromatid is the predominant repair template because of the close proximity of sister chromatids maintained by sister-chromatid cohesion [1], [2], and inter-sister recombination provides an important high fidelity pathway for DSB repair.

Meiosis is a specialized cell cycle in which diploid progenitor cells divide to produce haploid gametes [3], . The chromosome copy number is reduced in meiosis during the reductional division, in which homologous chromosomes are bioriented at metaphase to ensure that each daughter cell receives a haploid complement of chromosomes. The correct biorientation of homologous chromosomes generally requires the formation of physical linkages between homologous chromosomes called chiasmata, which are formed by reciprocal chromatid exchanges between homologous chromosomes that can occur through inter-homolog recombination. To promote chiasmata formation in meiosis I, a germ cell will purposely create up to hundreds of programmed DSBs which are repaired by HR [5]. While a small subset of DSB is repaired to form chiasmata, it is generally thought that the remaining DSBs must be efficiently repaired to preserve the genomic stability of the germ cell.

Even though homologous recombination between sister chromatids will not contribute to chiasmata formation, sister-chromatid recombination is responsible for a portion of meiotic DSB repair in a variety of species [6]–[11], and is thought to promote genomic stability in germ cells especially when inter-homolog recombination is compromised or unavailable [7], [10], [12]–[14]. However, whether meiotic sister-chromatid recombination is crucial for germ cell genomic stability, when inter-homolog repair is functional, has not been determined.

The *C. elegans* homolog of the mammalian breast and ovarian cancer susceptibility gene *brc-1* was implicated specifically for meiotic sister-chromatid recombination [7]. The *brc-1(tm1145)* mutant showed a delayed progression in HR repair, but there were no significant changes in chiasmata formation [7]. The *brc-1* mutation appeared to impede homolog-independent repair, because it caused the appearance of chromosome fragments when combined with mutations that disrupted inter-homolog repair [7]. However, the *brc-1* mutant by itself only exhibited a mild chromosome fragmentation defect [7]. These results suggest that *brc-1* and by extension sister-chromatid recombination are not required to complete meiotic DSB repair. The degree to which sister-chromatid recombination was disrupted by the *brc-1* mutation is unclear.

If sister-chromatid recombination in meiosis were necessary for the proper repair of meiotic DSB, then mutations that disrupted meiotic sister-chromatid recombination could result in chromosome anomalies (*e.g.* dicentric chromosomes), which could lead to mis-segregation and aneuploidy [15], [16]. Because mis-segregation and aneuploidy in meiosis also are the expected outcomes from the loss of inter-homolog recombination, it would be difficult to distinguish an inter-sister recombination defect from an inter-homolog recombination defect. The study of sister-chromatid recombination in meiosis would be simpler in an experimental organism, in which the presence of chromosome fragments and rearrangements would not necessarily lead to mis-segregation. One such model organism is the nematode *Caenorhabditis elegans* that can segregate partial chromosome duplications and fusion chromosomes with surprisingly high fidelity [17], [18]. We hypothesized that severe DSB repair defects in sister-chromatid recombination could be decoupled from chromosome mis-segregation when studied in *C. elegans*.

For a candidate meiotic sister-chromatid recombination factor, we chose the Structural Maintenance of Chromosomes (SMC) 5 and 6 protein complex, because the human and yeast Smc5/6 complexes have previously been implicated in sister-chromatid recombination in mitosis [19]–[21]. The mouse and fission yeast Smc5/6 protein complexes also are expressed during meiosis and the loss of the fission yeast complex leads to aneuploid spore formation [22], [23]. However, the requirement for the Smc5/6 complex specifically for meiotic sister-chromatid recombination has not been addressed.

The main objective of this research is to address the requirement and the function of the *C. elegans* SMC-5 and SMC-6 proteins for DSB repair in meiotic germ cells. The *C. elegans smc-5* and *smc-6* mutants exhibited defects in the processing of RAD-51 HR intermediates in meiosis. Similar to the *brc-1* mutant, chiasmata formation and meiotic chromosome segregation were apparently unaffected in the *smc-5* and *smc-6* mutants. In this study, we demonstrate that the RAD-51 defect is due to homolog-independent repair. More importantly, we find that the severe loss-of-function mutation in *smc-5* or *smc-6* is sufficient to elicit a chromosome fragmentation defect in meiotic germ cells. The fragments appeared to be associated with homolog-independent repair of programmed meiotic DSBs. Consistent with a loss in sister-chromatid recombination, the *smc-5* and *smc-6* mutant fragmentation defect was enhanced by inter-homolog recombination mutations, but not by mutations that reduced sister-chromatid recombination or cohesion. While the *smc-5* and *smc-6* mutants were initially viable, the mutant strains would gradually lose fecundity and exhibit other germ cell defects. These results reveal that the SMC-5/6 proteins function in homolog-independent, likely sister-chromatid-mediated, recombination in meiosis, and that homolog-independent recombination is required for germ cell genomic stability.

## Results

### The identification of the *C. elegans smc-5* and *smc-6* deletion mutants and the production of specific antibodies to the SMC-5 and SMC-6 proteins

**Figure 1:**
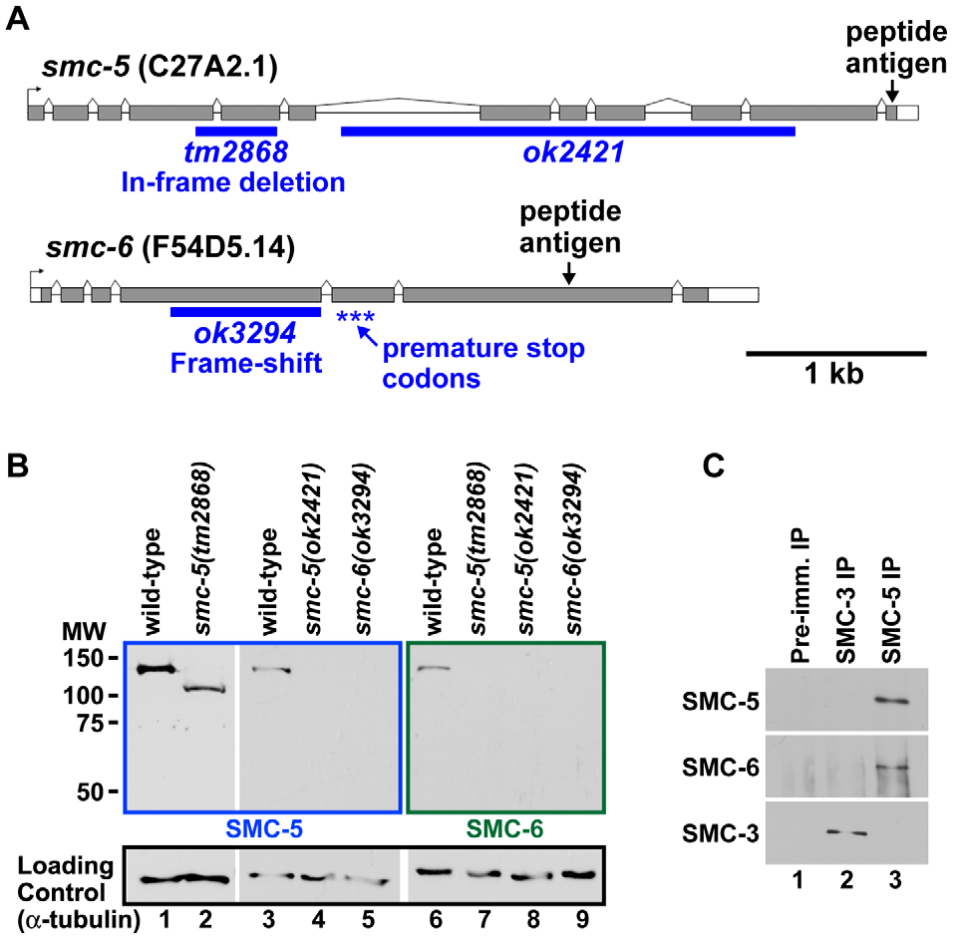
Identification and detection of the SMC-5 and SMC-6 homologs in *C. elegans*. (A) Two diagrams illustrate the predicted exon-intron structures of the *smc-5* and *smc-6* genes and the locations of the *tm2868*, *ok2421* and *ok3294* deletion mutations (blue bars). The exons are represented by the grey boxes and the introns by the black lines. The *tm2868* deletion removes 444 basepairs from exon 4 to exon 5, and it is predicted to generate an in-frame deletion of 133 codons. The *ok2421* deletion removes 2,420 basepairs from intron 6 to exon 11. The *ok3294* lesion deletes the last 830 basepairs from exon 4, and should result in a frame-shift at codon 192 and the introduction of premature termination codons (asterisks). The corresponding peptide regions used for antibody production are indicated. (B) Western blot analysis of whole worm lysates using antibodies raised to SMC-5 (lanes 1–5), antibodies to SMC-6 (lanes 6–9), and antibodies to alpha-tubulin as a loading control (lanes 1–9). The genotypes of the worms used are indicated above the western blot images. Two-fold more lysates were loaded in lanes 1–2 to detect the smaller SMC-5 product from the *smc-5(tm2868)* mutant lysates. (C) Western blot analysis of immunoprecipitates from wild-type embryo lysates using pre-immune (lane 1), SMC-3 (lane 2) and SMC-5 antibodies (lane 3). Antibodies for western blot detection are indicated to the left of the western blot images.

The *C. elegans* SMC-5 homolog C27A2.1 and the SMC-6 homolog F54D5.14 were identified previously based on protein sequence homology [24]. We generated antibodies to the SMC-5 and SMC-6 homologs that detected proteins of the predicted size from wild-type worm lysates (Figure 1B, lanes 1, 3 and 6). Western blot analyses of the mutant worm lysates confirmed the specificities of the antibodies, because the detected bands were absent in the *smc-5(ok2421)* and *smc-6(ok3294)* mutants (Figure 1B, lanes 4 and 9) that were predicted to have severe disruptions in protein function (Figure 1A). The SMC-5 antibodies also detected a smaller protein band in the *smc-5(tm2868)* mutant (Figure 1B, lane 2), in agreement with the predicted in-frame deletion in the *smc-5(tm2868)* encoded protein (Figure 1A). Immunoprecipitation analysis revealed that the *C. elegans* SMC-5 and SMC-6 proteins specifically co-precipitated from whole worm lysates (Figure 1C), as expected from the known association between the Smc5 and Smc6 proteins in yeast and in human [23], [25]–[27].

### The SMC-5 and SMC-6 proteins are enriched in the adult germline

**Figure 2:**
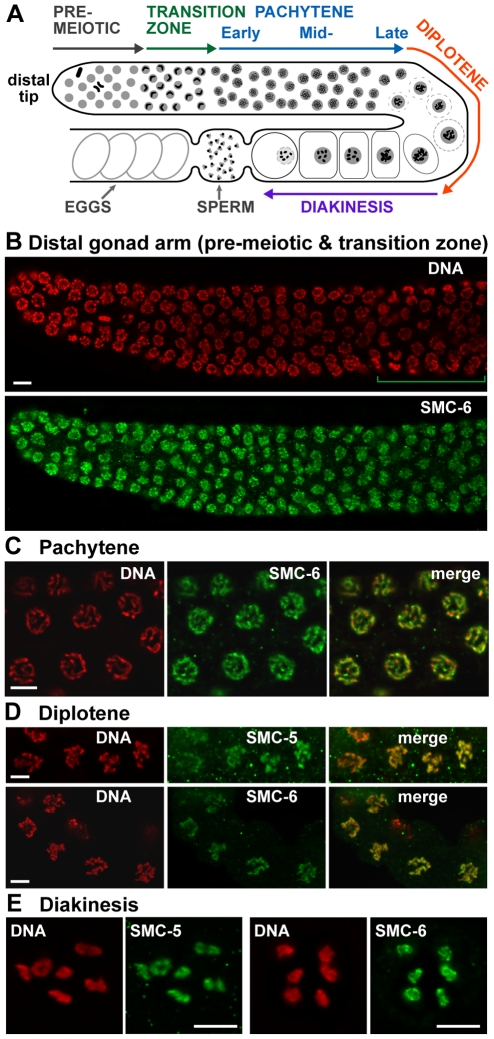
SMC-5 and SMC-6 accumulate in germ cells and on meiotic chromosomes. (A) A drawing representation of an adult hermaphrodite gonad arm. The progression of germ cell proliferation and meiosis are indicated by the arrows starting from the distal tip region of the gonad arm. (B) SMC-6 staining was detected in the nucleus of germ cells in the distal gonad arm. Mitotic germ nuclei are located in the pre-meiotic region proximal to the distal tip. Pre-meiotic S phase nuclei should be located proximal to the transition zone nuclei [66]. The green bracket marks the transition zone region. (C) SMC-6 staining in pachytene nuclei appeared to accumulate on chromosomes, which differed from the more diffuse nucleoplasmic staining in the distal gonad arm in (B). In the diplotene (D) and diakinesis (E) stages of meiotic prophase I, SMC-5 and SMC-6 staining were detected exclusively on chromosomes. Scale bars = 5 µm.

The SMC-6 protein was detected by indirect immunofluorescence microscopy in the nuclei of germ cells throughout the adult hermaphrodite gonad (Figure 2). Beginning at the distal tip region of the gonad (Figure 2A), SMC-6 staining is detected in the nucleus of germ cells in mitotic proliferation, pre-meiotic S phase and in the early stages of meiosis (the transition zone), which are equivalent to leptotene and zygotene (Figure 2B). Interestingly, SMC-6 staining became more enriched on chromosomes at pachytene (Figure 2C), which coincided with the timing of meiotic DSB repair [14]. The SMC-6 immunofluorescence became more intense as the germ cells exited pachytene and progressed through the diplotene and diakinesis stages of prophase (Figures 2D and 2E). The pachytene and diakinesis staining of SMC-6 was specifically disrupted by the *smc-6(ok3294)* mutation (Figures S1C and S1E). Even though we could not detect SMC-5 immunostaining on pachytene chromosomes, we found that the *smc-5(tm2868)* and *smc-5(ok2421)* mutations reduced SMC-6 staining on pachytene chromosomes (Figures S1A and S1B), indicating a possible dependency on SMC-5 for the localization of SMC-6 at pachytene. The immunostaining for SMC-5 was detected on diplotene and diakinesis chromosomes specifically in wild-type (Figures 2D and 2E), but not in the *smc-5(tm2868)* and *smc-5(ok2421)* mutant oocytes (Figures S1D and S1H). The SMC-5 and SMC-6 chromosomal staining in diplotene (data not shown) and diakinesis oocytes (Figures S1F, S1G and S1I) also appeared to be interdependent. In addition to germ cell staining, SMC-5 and SMC-6 immunostaining were detected in somatic cells during early embryogenesis (data not shown). The three *smc-5* and *smc-6* mutations caused frequent chromatin-bridges to appear in the intestine, even though immunostaining was significantly weaker in the intestine than the germline (Bickel and Chan, unpublished observations). These results suggest that the SMC-5/6 proteins accumulate in the soma and the germline, with greater enrichment seen in the germ cells.

### Mutants of *smc-5* and *smc-6* exhibited reduced fecundity

The homozygous *smc-5* and *smc-6* mutants (F1) produced by heterozygous mutant parents and the F2 offspring produced by the homozygous *smc-5* and *smc-6* F1 mutants exhibited near wild-type embryonic viability (98% to 99% viable with n>2,000 per genotype). Since the F2 mutants lacked maternal and zygotic expression of the SMC-5 or the SMC-6 protein, we conclude that the *C. elegans* SMC-5 and SMC-6 proteins are dispensable for viability. However, the *smc-5* and *smc-6* mutants were difficult to maintain as homozygous mutant strains and were prone to becoming sterile (Figure S2A). The transgenerational sterility phenotype is typically associated with genomic instability in the germ cells [28]–[30]. By contrast, the homozygous *brc-1(tm1145)* mutant strains remained fecund (Figure S2A), which suggests the *smc-5* and *smc-6* mutations may be more disruptive to normal germ cell functions.

In agreement with the observed enrichment for the SMC-5 and SMC-6 proteins in germ cells, the *smc-5* and *smc-6* mutants had significantly smaller gonads containing fewer germ cells (Figure S3). In comparison to wild-type, there was on average a 30% to 50% reduction in fertilized eggs produced by the *smc-5* and *smc-6* F1 mutants and in *smc-5* RNAi-treated worms (Figure S2B). Figure S2C shows that the *smc-5(tm2868)* mutant and the wild-type worms both began to lay eggs at approximately 1 day after L4 development. However, fertilized egg production declined in the *smc-5(tm2868)* mutant at an earlier age than wild-type (Figure S2C) and the mutant hermaphrodites began to lay unfertilized oocytes (data not shown). Similar phenotypes were also observed for the *smc-5(ok2421)* and the *smc-6(ok3294)* mutants (data not shown). The *C. elegans* hermaphrodite produces and stores a supply of sperm during late larval development, which will be used to fertilize oocytes produced in adulthood. The appearance of unfertilized oocytes could be caused by a premature depletion of hermaphrodite-produced sperm. The *smc-5(tm2868)* mutant hermaphrodites indeed showed an absence of sperm at an earlier age in adulthood (Figure S2D). However, male sperm supplied by mating failed to restore egg production (Figure S2E), which indicates that the loss of fecundity is not simply due to an insufficient amount of sperm.

### Germ cells from the *smc-5* and *smc-6* mutants exhibited phenotypic defects in DNA damage repair–hypersensitivity to ionizing radiation and increased germ cell apoptosis

**Figure 3:**
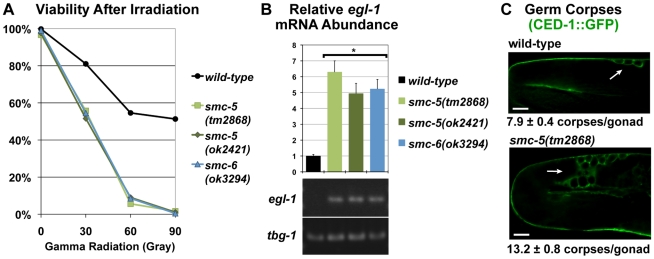
Loss of function mutations in *smc-5* and *smc-6* conferred hypersensitivity to ionizing radiation (IR) and increased DNA-damage responses in germ cells. (A) Graph of the viability of the eggs produced from mock and radiation-exposed germ cells in mutant F1 and wild-type L4-stage larvae as described [31]. 297 eggs or more were counted for each genotype and at each dose of IR. (B) Relative mRNA abundance for *egl-1* after normalization to gamma tubulin *tbg-1* as measured by quantitative RT-PCR [32]. Error bars represent standard errors. The asterisks (*) indicate significant changes from the wild-type control (p<0.001, t-Test). Gel analysis confirmed the size of each RT-PCR product and is shown underneath the graph. (C) Micrographs of germ cell corpses (white arrows) detected by CED-1::GFP fluorescence showed a greater number of corpses in the *smc-5(tm2868)* mutant germline. The average number of corpses per gonad and the standard error are indicated. Scale bars = 10 µm.

In order to determine if the *smc-5* and *smc-6* mutants have defects in the maintenance of germ cell genomic stability, we tested whether germ cells lacking SMC-5/6 functions are hypersensitive to ionizing radiation (IR). We followed a published protocol that examines the viability of eggs produced from radiation-damaged germ cells [31]. For each dose of gamma radiation exposure examined, the *smc-5* and *smc-6* mutants exhibited drastically reduced viability in comparison to wild type, consistent with the mutant germ cells being hypersensitive to DNA damage (Figure 3A).

Because the *smc-5* and *smc-6* F1 mutants showed the aforementioned gonadal defects (Figures S2 and S3) even before IR treatment, we examined whether DNA damage may already be present in the germline of unchallenged *smc-5* and *smc-6* mutants. A well-characterized DNA damage response in the *C. elegans* hermaphrodite germline is the induction of apoptosis by CEP-1/p53, via the transactivation of the pro-apoptotic gene *egl-1* [32]. Quantitative RT-PCR analysis showed a 5- to 6-fold increase in *egl-1* transcript levels in the *smc-5* and *smc-6* mutant worms in comparison to wild type (Figure 3B). There was also an approximate two-fold increase in germ cell corpses in all three *smc-5* and *smc-6* mutant strains as detected by acridine orange staining (Table S1). This staining was specifically suppressed in the *smc-5(tm2868);ced-3(n717)* double mutant, which had an impaired ability to implement apoptosis (Table S1). The germ cell corpses also were confirmed in the *smc-5(tm2868)* mutant using a CED-1::GFP reporter that marked cell corpses during endocytosis (Figure 3C and Table S2). The RNAi knockdowns of three core pro-apoptotic genes *ced-3*, *ced-4*, and *egl-1* suppressed the appearance of the GFP-positive germ cells in the *smc-5(tm2868)* mutant, indicating that they were apoptotic germ cell corpses (Table S2). In summary, the molecular, cytological and functional evidence demonstrated that the *smc-5* and *smc-6* mutations resulted in increased DNA damage response.

### The *smc-5* and *smc-6* mutants showed an abnormal accumulation of SPO-11-dependent homologous recombination intermediates

We next examined whether meiotic DSB repair was compromised in the *smc-5* and *smc-6* mutant germ cells, by monitoring the appearance and disappearance of the HR strand exchange protein RAD-51 on meiotic chromosomes [33]. DNA DSBs are formed by the SPO-11 topoisomerase-like proteins at meiosis entry [34]. Following 5′-to-3′ single-strand end resection, the RAD-51 proteins localize to the single-stranded DNA to promote the invasion of intact homologous DNA template [14], [33]. RAD-51 focal formation usually reaches a maximum density (foci per nucleus) at early-pachytene and RAD-51 foci begin to disappear at mid-pachytene [14], [35], [36]. By late pachytene, there are few RAD-51 foci as HR repair is completed.

For comparison of equivalent gonadal regions between the wild-type and the mutant strains, we limited our analysis of RAD-51 focal staining to mutant gonad arms that were close in size to wild type. RAD-51 staining appeared in the distal region of the *smc-5* and *smc-6* mutant gonads, where it is rarely seen in wild-type (Figures S4A–S4C); the quantification of the results is shown in Figure S4F. The germ cells at the distal region are in mitosis and pre-meiotic DNA replication, and therefore prior to meiotic entry. We suspected that these RAD-51 foci were independent of meiotic DSB. In agreement with this prediction, we found that the RAD-51 foci in the distal gonads of both *smc-5* mutant alleles (*tm2868* and *ok2421*) persisted even though meiotic DSB formation was blocked by the *spo-11(ok79)* null mutation [34] (Figures S4D and S4E). The budding and fission yeast Smc5/6 mutants are known to exhibit Rad51-dependent DNA replication defects [20], [37]–[42]. The abnormal RAD-51 staining seen here in the pre-meiotic germline in *C. elegans* may represent a DNA replication defect.

**Figure 4:**
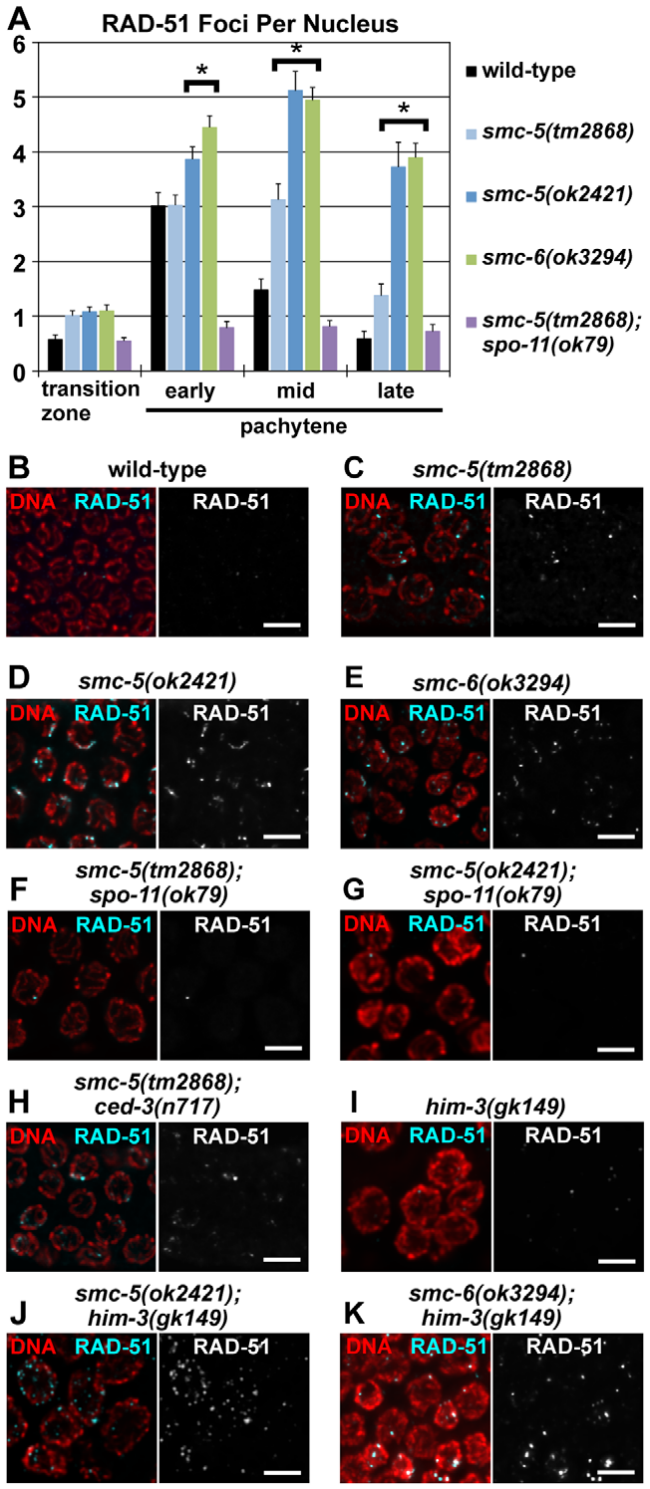
Aberrant RAD-51 accumulation in meiotic germ cells from the *smc-5* and *smc-6* mutants. (A) The average numbers of RAD-51 foci per nucleus in the transition zone and pachytene regions of the gonad arms are represented in the bar graph; the error bars represent standard errors. The pachytene region was divided into three equal length areas (early, mid and late) and each quantified separately. The *smc-5* and *smc-6* mutants showed significant increases in RAD-51 foci in comparison to wild-type at the mid- to late-pachytene regions (p≤0.01; two-tailed t-Test). The numerical values and sample sizes are summarized in Table S3. (B–K) Micrographs of RAD-51 immunofluorescence and DAPI-DNA fluorescence in late pachytene nuclei from the wild-type and the mutant germlines. Scale bars = 5 µm.

In the transition zone and at early pachytene, the meiotic germ cells exhibited slightly more RAD-51 foci in the *smc-5* and *smc-6* mutants than in wild-type (Figure 4A). The difference in the number of RAD-51 foci between the mutants and the wild-type was significantly more pronounced at mid- and late-pachytene (Figures 4A–4E). In contrast to the pre-meiotic RAD-51 staining, the aberrant pachytene RAD-51 staining in the *smc-5(tm2868)* and the *smc-5(ok2421)* mutants was significantly reduced by the *spo-11(ok79)* mutation (Figures 4F and 4G), which suggests that the RAD-51 staining defect at pachytene resulted primarily from a defect in meiotic DSB repair rather than from prior DNA damage in the pre-meiotic region.

Because apoptosis in germ cells could generate corpses that have elevated RAD-51 staining, we also examined the *smc-5(tm2868);ced-3(n717)* double mutant that was deficient for germ cell apoptosis. The *ced-3* mutation failed to suppress the aberrant RAD-51 staining at late pachytene in the *smc-5(tm2868)* mutant, thus ruling out the possibility that the RAD-51 defect was induced by apoptosis (Figure 4H).

Homologous recombination appears to be the predominant pathway for meiotic double-strand break repair in *C. elegans* [43], [44]. The *lig-4(ok716)* mutant in the canonical non-homologous end-joining (NHEJ) pathway has no measurable effects on RAD-51 accumulation in the germline [10]. Therefore, the aberrant RAD-51 staining in the *smc-5* and *smc-6* mutants likely reflects a defect in homologous recombination repair rather than NHEJ.

### SMC-5 and SMC-6 are dispensable for inter-homolog crossover formation

**Figure 5:**
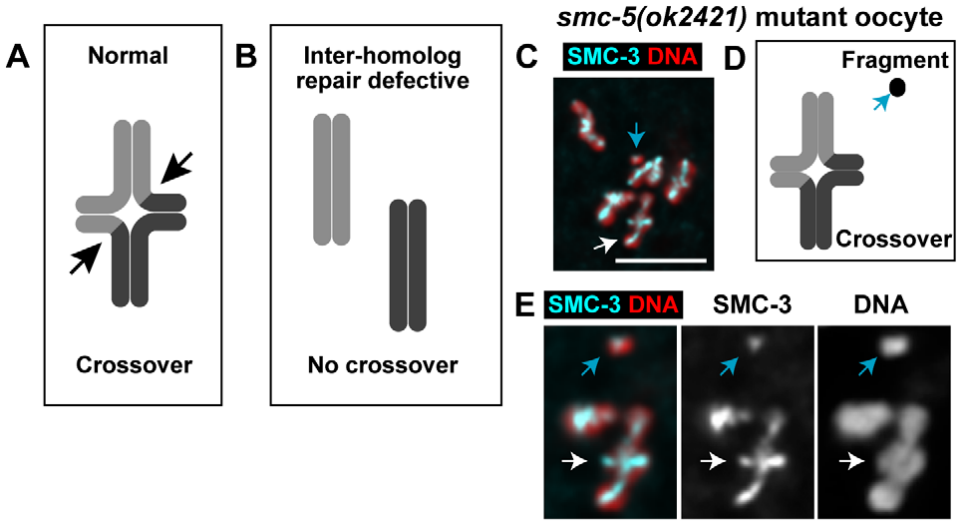
Chromosome fragmentation defects in *smc-5* and *smc-6* mutant diakinesis oocytes that successfully formed crossovers. Cartoon representations (A and B) of a pair of homologous chromosomes at diakinesis. (A) In wild-type, inter-homolog association is maintained by cohesion between the recombinant chromatids. The location of the chromatid exchange is indicated by the two black arrows, which provides the focal point for the restructuring of chromatid axes that results in a “cross-shape” or cruciform structure of the diakinesis chromosomes in *C. elegans* [67]. (B) If inter-homolog crossover failed to form, the homologous chromosomes would prematurely separate and the chromosome axes will not have the cruciform shape. (C) Micrograph of an *smc-5(ok2421)* mutant oocyte containing six linked homolog pairs with the wild-type cruciform shape, indicating successful inter-homolog crossover formation. Projection depth and scale bar = 5 µm. (D) A cartoon representation of the linked homolog and DNA fragment shown in (C and E). (E) Magnified view of a linked homolog pair (white arrow) and a chromosome fragment (light blue arrow) from (C) for which cohesin SMC-3 staining also was detected on the fragment. Projection depth = 2 µm.

The failure to efficiently form inter-homolog crossover and the subsequent chromosome mis-segregation in meiosis would produce two expected phenotypes: low embryonic viability due to aneuploidy and higher than normal frequency of XO males due to X chromosome mis-segregation. The *smc-5* and *smc-6* F2 mutants exhibited normal embryonic viability (98% to 99%) and wild-type frequencies of male self-progeny (0.1% to 0.2%). Chiasmata formation appeared to be normal in the *smc-5* and *smc-6* mutant oocytes, because the mutant oocytes had an average of six DNA figures per oocyte consistent with normal inter-homolog linkage from the six pairs of homologous chromosomes (Table S4). We also observed the restructuring of meiotic chromosomes at late prophase consistent with chiasmata formation (Figure 5), as confirmed by cohesin immunostaining and DNA-DAPI fluorescence of the chromatid axes (Figures 5C–5E). The ZHP-3 proteins are proposed to couple synaptonemal complex morphogenesis and crossover formation and to mark precursor sites for inter-homolog crossover formation in late pachytene [45], [46]. The average number of ZHP-3 foci remained unchanged at the wild-type level of six foci per nucleus in the *smc-5(ok2421)* and *smc-6(ok3294)* mutant germ cells (Figure S5; [45]). Thus, the combination of phenotypic and cytological evidence confirmed inter-homolog crossover formation in the *smc-5* and *smc-6* mutant germlines.

### The *smc-5* and *smc-6* mutations appeared to disrupt homolog-independent homologous recombination in meiosis

**Figure 6:**
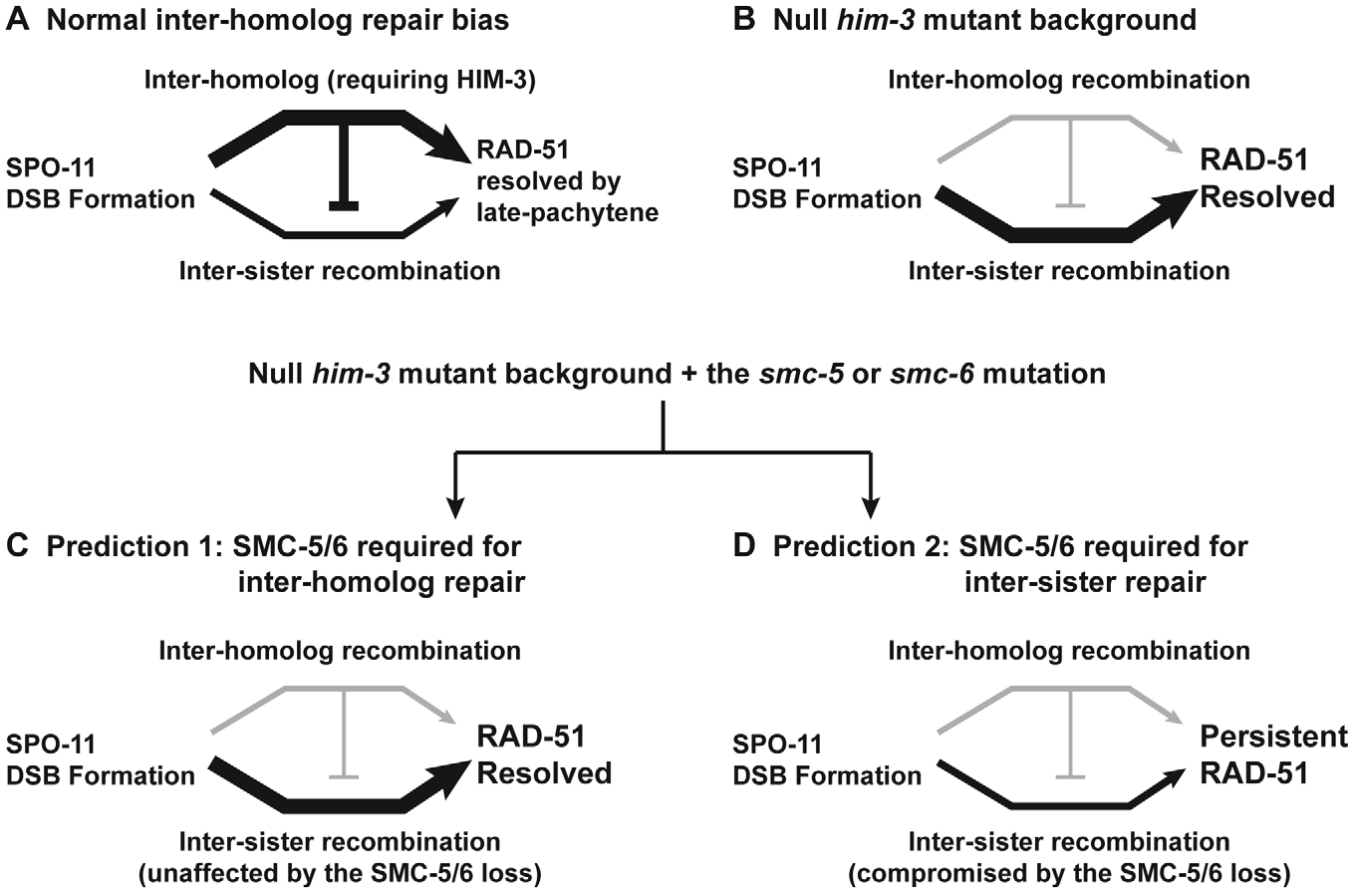
Possible outcomes for the combined genetic disruptions of *smc-5* or *smc-6* and *him-3*. (A) Schematic diagram depicts the biased repair of SPO-11 DSBs via inter-homolog recombination repair in meiosis. (B) In the absence of HIM-3, SPO-11 DSBs are repaired via homolog-independent (*i.e.* sister-chromatid) recombination [48]. (C–D) Two predicted repair outcomes for the double mutants. (C) If the SMC-5/6 proteins function mainly in inter-homolog repair which is already disrupted by the *him-3* mutation, then the overall repair efficiency as monitored by the removal of RAD-51 staining should be the same in the double mutants as compared to the *smc-5* and *smc-6* single mutants. (D) If the SMC-5/6 proteins function mainly in inter-sister repair, then the double mutant should exacerbate the repair defects and the RAD-51 intermediates should persist at late pachytene.

Based on our findings, the SMC-5/6 proteins appear to be required for homologous recombination that does not affect inter-homolog crossover formation, which possibly involves inter-homolog non-crossover repair or homolog-independent sister-chromatid recombination. To address the two possibilities, we tested whether the *smc-5(ok2421)* and the *smc-6(ok3294)* mutations will confer the RAD-51 defect in the homolog synapsis mutant *him-3(gk149)*. HIM-3 is an axial element of the synaptonemal complex that promotes inter-homolog recombination by stabilizing the close association of homologous chromosomes in wild-type (Figure 6A; [47]). In the absence of HIM-3 (Figure 6B), inter-homolog non-crossover repair should be disrupted. If the *smc-5* or the *smc-6* mutation also disrupted inter-homolog non-crossover repair, then the *him-3* double mutant with *smc-5* or *smc-6* should be similar to the *him-3* single mutant (Figure 6C). Conversely, if the SMC-5/6 proteins promote homolog-independent recombination, which should be functional in the *him-3* mutant [48], then the RAD-51 staining in the *him-3* double mutant should be more persistent than the *him-3* single mutant (Figure 6D).

The RAD-51 focal staining at late pachytene in the *him-3(gk149)* null mutant was similar to wild-type (Figure 4I; [48]). The *smc-5(ok2421)* or the *smc-6(ok3294)* mutation in the *him-3* mutant genetic background drastically increased RAD-51 focal staining at late pachytene (Figures 4J and 4K), indicating that the *smc-5* and *smc-6* mutations impeded homolog-independent homologous recombination repair in meiosis.

### The *smc-5(ok2421)* and the *smc-6(ok3294)* mutants exhibited chromosome fragmentation and dismorphology at diakinesis

**Figure 7:**
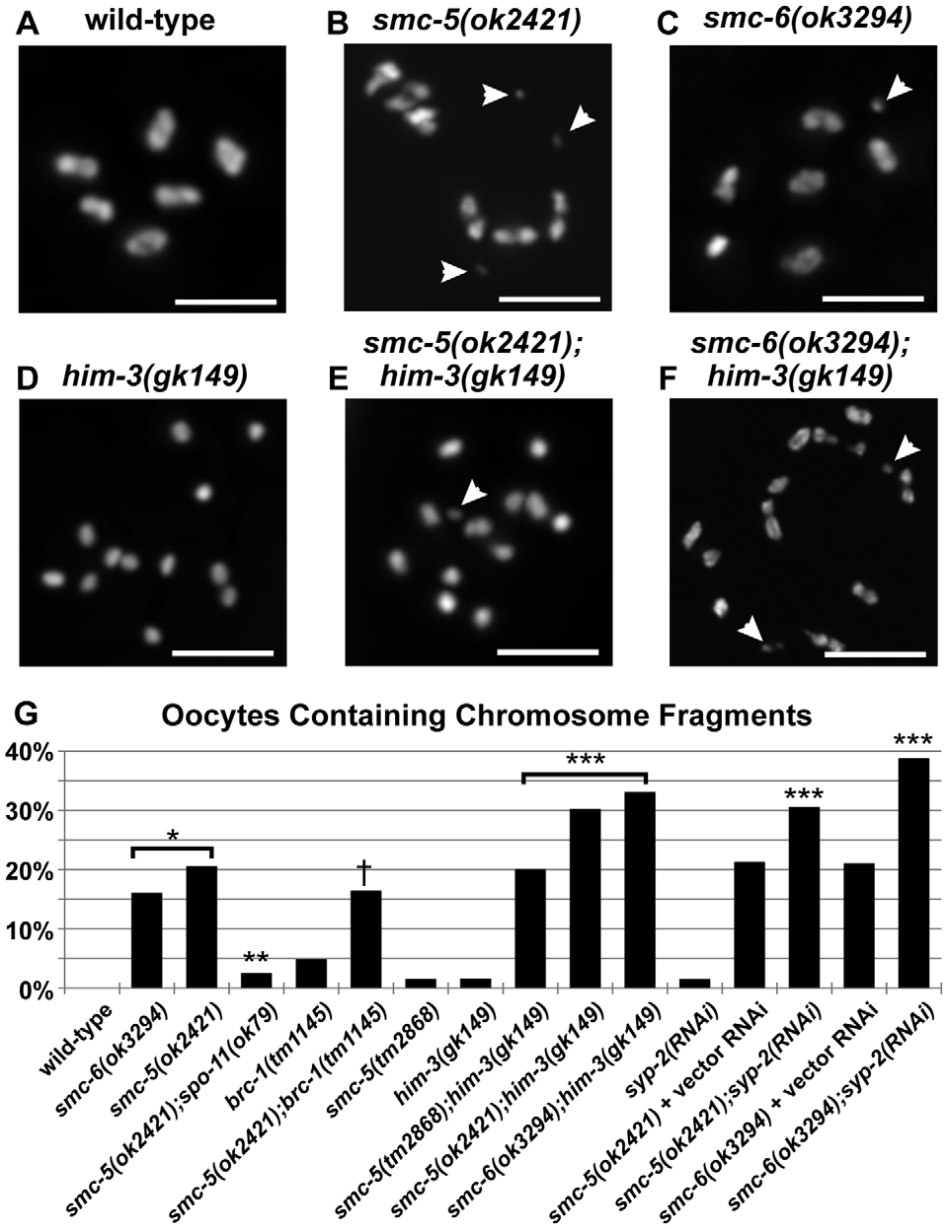
Chromosome fragmentation defects linked to homolog-independent repair of meiotic DSB. (A–F) Micrographs of DAPI-stained diakinesis chromosomes are shown at the same magnification. Chromosomal fragments are indicated by white arrowheads, and scale bars = 5 µm. (G) A graph of the percentages of diakinesis oocytes containing chromosome fragments. The *smc-5(ok2421)* and the *smc-6(ok3294)* mutant oocytes showed significantly higher frequency of chromosome fragmentation over wild-type (*p<0.001, Fisher's Exact Test). The *smc-5(ok2421)* fragmentation defect was drastically suppressed by the *spo-11(ok79)* mutation (**p = 0.006). The disruption of inter-homolog recombination by destabilizing homolog-synapsis consistently enhanced the fragmentation defects in all three *smc-5* and *smc-6* mutant strains (***). By contrast, the *brc-1* mutation affecting sister-chromatid recombination [7] failed to enhance the fragmentation defect of the *smc-5(ok2421)* mutation (†). The numerical values, sample sizes, and statistical comparisons are summarized in Table S5.

Approximately 20% of the diakinesis oocytes from the *smc-5(ok2421)* and *smc-6(ok3294)* mutants contained chromosome fragments (blue arrows in Figure 5, and white arrowheads in Figure 7). These fragments were disproportionately smaller than the linked homologs at diakinesis, and also showed staining for the cohesin SMC-3 protein indicating they were derived from chromosomes (Figure 5E). By contrast, no fragments were observed in wild-type oocytes (Figures 7A and 7G). Intriguingly, the fragmentation defect was rarely seen in the *smc-5(tm2868)* mutant oocytes and appeared to correlate with the severity of SMC-5 and SMC-6 protein disruption because the *smc-5(tm2868)* mutants still produced a truncated SMC-5 protein (Figure 1B). The fragmentation defect also correlated with the more severe RAD-51 staining defect at late pachytene that was found in the *smc-5(ok2421)* and *smc-6(ok3294)* mutants (Figure 4A). Similar to the RAD-51 defect, the *spo-11(ok79)* null mutation potently reduced fragmentation from 20.5% in *smc-5(ok2421)* mutant oocytes to 2.4% in the *smc-5;spo-11* double mutant oocytes (Figure 7G).

The *brc-1(tm1145)* mutant reportedly exhibited a low penetrance fragmentation defect [7], which prompted us to compare the fragmentation defect in the *brc-1(tm1145)* mutant oocytes to the *smc-5* and *smc-6* mutants. There was an approximate three- to four-fold lower incidence of the fragmentation defect in the *brc-1(tm1145)* mutant oocytes in comparison to the *smc-6(ok3294)* and the *smc-5(ok2421)* mutants, respectively (Figure 7G). The *smc-5* and *smc-6* mutant oocytes also exhibited an unresolved diakinesis chromosome defect that was absent in the *brc-1(tm1145)* mutant and the wild-type oocytes (Figures S6A and S6B). Unresolved diakinesis chromosomes often appeared with a severe loss of homologous recombination function as found in the *rad-51(lg8701)* and other HR repair mutants (Figure S6A; [14],[33],[44],[49]). These results together indicate that the *smc-5(ok2421)* and the *smc-6(ok3294)* mutants may be more severely compromised for meiotic homologous recombination repair than the *brc-1(tm1145)* mutant.

We utilized chromosome fragmentation at diakinesis as a quantitative readout to determine the genetic interactions of *smc-5* and *smc-6* with inter-homolog recombination mutations or sister-chromatid recombination and cohesion mutations. If the fragmentation defect of the *smc-5* and *smc-6* mutants was due to disruptions in sister-chromatid recombination, then additional mutations that disrupted inter-homolog recombination (*e.g.* homolog-synapsis mutants) should enhance the fragmentation defect. Moreover, additional mutations that disrupted sister-chromatid recombination or cohesion would be expected to not enhance the fragmentation defect. We examined two homolog-synapsis defective conditions, using the *him-3(gk149)* null mutation and RNAi knockdown of *syp-2*, which encodes a synaptonemal complex central element protein [14]. While the loss of function for *him-3* or *syp-2* in the wild-type genetic background caused a modest chromosome fragmentation defect (1.4% to 1.5%), the homolog synapsis deficiencies consistently enhanced the penetrance of the fragmentation defects in the *smc-5(ok2421)* and the *smc-6(ok3294)* mutants. There were synergistic increases from an average of 20% fragmentation for the *smc-5(ok2421)* and *smc-6(ok3294)* single mutants to 30% to 39% for the double mutants (Figure 7G). This enhancement was more pronounced in the sensitized *smc-5(tm2868)* hypomorphic mutant. The *smc-5(tm2868);him-3(gk149)* double mutant oocytes exhibited chromosome fragments in 20% of the oocytes, which was a 10-fold increase in fragmentation compared to the *smc-5(tm2868)* and the *him-3(gk149)* single mutants (Figure 7G). By contrast, double mutants with *brc-1(tm1145)* (Figure 7G) or the cohesin *Smc1* mutant, *him-1(e879)*, failed to enhance the fragmentation defect (Figure S6C). The *brc-1(tm1145)* and the *him-1(e879)* mutations disrupt sister-chromatid recombination and cohesin function, respectively [7], [50]. Thus, the specific enhancements of the fragmentation defect by the loss of homolog-synapsis and not by further disruptions to sister-chromatid recombination support the hypothesis that the SMC-5/6 proteins function in meiotic sister-chromatid recombination.

## Discussion

### The successful completion of homolog-independent homologous recombination is crucial for genomic stability in germ cells

The requirement for homolog-independent recombination under normal meiotic conditions, when inter-homolog repair is functional, has not been tested. Challenges in addressing this question are the lack of experimental means to robustly disrupt homolog-independent recombination in meiosis without also perturbing inter-homolog recombination.

This study provides evidence that the SMC-5/6 proteins in *C. elegans* are required for the successful completion of homologous recombination repair of meiotic DSBs, consistent with similar observations in fission yeast [22]. Our findings further pinpoint this requirement for the SMC-5/6 proteins in *C. elegans* to homolog-independent homologous recombination. The *smc-5* and *smc-6* mutations disrupted the normal progression of homolog-independent homologous recombination intermediates in the *him-3(gk149)* mutants (Figures 4J and 4K). Although we have not directly assessed sister-chromatid recombination in the meiotic germ cells, the genetic interactions between *smc-5(ok2421)* and *brc-1(tm1145)* (Figure 7G) and between *smc-5(ok2421)* or *smc-6(ok3294)* with the *him-1(e879)* mutation (Figure S6C) are consistent with sister-chromatid recombination being disrupted in the *smc-5(ok2421)* and *smc-6(ok3294)* mutants. Additionally, the *smc-5(ok2421)* and *smc-6(ok3294)* mutants each showed a gradual transgenerational sterility defect, consistent with the loss of germ cell genomic stability. Together, the cytological and phenotypic evidence from the *smc-5* and *smc-6* mutants provides the first experimental support that the proper execution of homolog-independent recombination in meiosis is crucial for genomic stability in germ cells.

### Chromosome fragmentation at diakinesis

A chromosome fragment arising simply from incomplete DSB repair in a chromatid should remain bound to its sister chromatid by cohesion and should not appear as a free fragment at diakinesis. Sister-chromatid cohesion is presumably maintained in the *smc-5* and *smc-6* mutants as evident by the intact chiasma and the cohesin SMC-3 staining on diakinesis chromosomes (Figure 5E and Table S4). Therefore, we hypothesize that the appearance of fragments may require the localized loss of chromatid cohesion near the site of homolog-independent repair in addition to incomplete or aberrant DSB repair. In budding yeast, cohesin-mediated repair in G2 phase of the mitotic cell cycle can establish *de novo* damage-induced cohesion [51], [52], and cohesin-mediated repair in both budding yeast and human requires the Smc5/6 complexes [19]–[21], [53]. Although evidence suggests that damage-induced cohesion may not form during meiosis in budding yeast [54], whether damage-induced cohesion exists in meiosis in other species remains to be determined. Intriguingly, the human Rec8 cohesin subunit that functions in meiosis was able to rescue damage-induced cohesion in the budding yeast *scc1(mcd1)* mutant [54].

As another possibility, the loss of Smc5/6 function could produce abnormal chromosome structures and fragments through ectopic recombination. The fission and budding yeast Smc5/6 complexes are thought to function in a late step in recombination repair, perhaps in the regulation of HR intermediates [20], [37], [55], [56]. Aside from DNA damage response, the yeast Smc5/6 complexes also are required for genomic stability during DNA replication. Budding and fission yeast mutants deficient for the Smc5/6 complexes were found to accumulate Rad51-dependent joint molecules near collapsed replication forks [37], [38]. These aberrant structures are thought to result from ectopic template switching events in the resuscitation of collapsed replication forks [38], [57]. A similar DNA replication defect in the *C. elegans smc-5* and *smc-6* mutant germ cells may account for the abnormal SPO-11-independent RAD-51 staining seen in the pre-meiotic region (Figure S4). This function may be analogous to restraining ectopic strand invasion events during homologous recombination repair. Such a defect during homologous recombination repair could explain the increased staining intensity for RAD-51 in the *smc-5* and *smc-6* mutant germ cells at pachytene. The proposed roles for the SMC-5/6 complex in damage-induced cohesion and the regulation of ectopic recombination may underlie meiotic defects not only in programmed DSB repair but also inappropriate cohesion loss near the DNA lesion, and the combination of both defects may account for the increased frequency for chromosome fragments at diakinesis.

### An experimental model to examine homolog-independent homologous recombination repair

We showed that chromosome fragmentation and dismorphology defects were present in the *smc-5* and *smc-6* mutant oocytes just prior to fertilization, but they had no discernable effects on offspring viability and chromosome segregation. This finding supports our hypothesis that *C. elegans*, as an experimental model, could decouple the successful completion of meiotic DSB repair from the potentially lethal effects of chromosome fragments and other abnormal chromosome configurations. *C. elegans* may be uniquely useful to study the molecular mechanisms involved in homolog-independent repair, which may be utilized in many species to maintain genomic integrity during meiosis [6]–[11].

## Materials and Methods

### Genetics and phenotypic analyses

The *C. elegans* Bristol *(N2)* strain served as the wild-type control and all strains were maintained at 20°C under standard growth conditions [58]. Genetic mutations, rearrangements and transgenic reporters used in this study are as follows:

LGI: *him-1(e879)* [50]

LGII: *smc-5(tm2868)*, *smc-5(ok2421)*, *smc-6(ok3294)* [In this study], *mIn1[dpy-10(e128) mIs14[myo-2::gpf; pes-10::gfp]]* [59]

LGIII: *brc-1(tm1145)* [7]

LGIV: *him-3(gk149)* [48], *spo-11(ok79)* [34], *rad-51(lg8701)* [33], *nT1[qIs51] (IV:V), nT1[unc-?(n754) let-?] (IV;V)* [60]

LGV: *bcIs39[lim-7::ced-1-gfp]* [32],[61]

RNAi knockdown for *smc-5* was induced by microinjection of 1–2 mg/mL of double-stranded RNA specific to the *smc-5* gene in phosphate-buffered saline (PBS) in adult hermaphrodites at 1 day after L4 development, and mock control worms were injected with PBS containing no RNA. The *smc-5* RNA was transcribed *in vitro* using T7 RNA polymerase (Promega) and a double-stranded DNA template containing T7 promoter sequences (underlined in lower case below) amplified from wild-type *N2* genomic DNA using the following primers:

5′ gcgtaatacgactcactatagggTCCGTGTGCATTTCTTGCTC 3′ and

5′ gcgtaatacgactcactatagggATAGGCTTTCGAGGCATCAC 3′

The RNAi knockdown of *syp-2* was performed as described [45].

For the germline apoptosis analysis, worm strains containing the CED-1::GFP reporter construct [32] were grown at 20°C until the L4 larval stage and were then shifted to 25°C for 16 hours prior to analysis for GFP-positive germ corpses.

For ionizing radiation hypersensitivity, L4 hermaphrodites from wild-type and F1 mutant strains were irradiated in a Philips RT250 orthovoltage unit at 30, 60 and 90 Gy of ionizing radiation. The worms recovered for 24 hours before eggs were collected and counted for embryo lethality as described [31]. 297 to 545 embryos were counted for each genotype per dose of radiation.

### Quantitative RT-PCR analysis

Approximately 500 embryos were collected and grown for 72 hours at 20°C to young adulthood (approximately 1 day after L4 larval development), at which time they were harvested for RNA extraction using TRIzol reagent (Invitrogen). 250 ng of extracted RNA were used for oligo(dT)-primed cDNA synthesis using Superscript III First Strand Synthesis Kit (Invitrogen). Quantitative PCR were performed using the ABI 7500 Fast Real Time PCR system and the SYBR Green PCR kit (5 Prime) following manufacturer's protocol but at reduced 15 µL reaction volumes. PCR primers for *egl-1* and gamma-tubulin *tbg-1* are described in [32]. All RT-PCR products were confirmed by melt curve analysis and by gel analysis to verify that the amplification depended on reverse transcription and the cDNA products were the expected size (Figure 3B). The amounts of template cDNA were titrated to validate the measurements. The mRNA abundance was calculated based on threshold cycle numbers (Ct) and normalized to *tbg-1* using the equation: relative abundance = 2^−[(Ct,gene-of-interest)−(Ct,normalization control)]^

### Antibody preparation, western blotting and immunoprecipitation

Rabbit antibodies were raised to peptides containing an amino terminal cysteine-glycine (CG) linker and the following sequences:

SMC-5 carboxyl terminus: TNSHGKHYDTSAKIDATFAKMGISA

SMC-6 internal residues 849–868: DAMEMVENDKKNHPMPPGET

Antibodies were purified on peptide affinity columns prior to use. Rat antibodies to *C. elegans* SMC-3 used for immunofluorescence were described in [50]. Commercial rabbit anti-human Smc3 antibodies (Bethyl Laboratories, Inc., cat. no. A300-060A) were used for the immunoprecipitation study. The cross-reactivity of anti-human Smc3 antibodies to the *C. elegans* SMC-3 protein was confirmed by western blot and mass spectrometry analysis (data not shown) of precipitated proteins. Other antibodies used in the study include mouse anti-tubulin DM1alpha (Sigma), mouse anti-p-granule OIC1D4 (Developmental Studies Hybridoma Bank, [62]) and guinea pig anti-ZHP-3 [45]. Rabbit anti-RAD-51 antibodies obtained from two different sources had nearly identical staining (Strategic Diagnostics, Inc., cat. no. 2948.00.02; and [33])

Worm lysate preparation, western blot and immunoprecipitation were performed as described [50], [63]. The detection of the SMC-5 and SMC-6 proteins in whole worm lysates required 50 to 100 wild-type worms, and 150 to 200 *smc-5(tm2868)* mutant worms for the truncated SMC-5 protein.

### Fluorescence microscopy

All micrographs were captured on an Olympus BX61 epifluorescence compound microscope with a Hamamatsu ORCA ER camera. Images of whole gonad arms were captured with a 10× Plan Fluorite (NA 0.3) dry objective. All other images were captured with a 60× Plan Apochromat (NA 1.35) oil objective at 0.25 to 0.28 µm z-sections and deconvolved using Huygens Essential software version 3.4 (Scientific Volume Imaging). Images were processed using ImageJ and Photoshop CS2 software packages. Slide preparation was performed as described [64] with the exception of ZHP-3 and RAD-51 (Strategic Diagnostics, Inc.) that were processed as described by [45]. Quantification of RAD-51 foci was performed on germline stained with the RAD-51 antibodies described by [33]. The intensity of RAD-51 fluorescence for individual gonads was normalized to the background fluorescence found in the rachis of the gonad. RAD-51 counts for the pre-meiotic nuclei were limited to the first eight rows of germ nuclei from the distal tip. The meiotic region of the germline was divided into four equal length sections from the beginning of transition zone (polarized chromosome morphology) to the exit from pachytene (onset of cellularization). The four sections were classified as transition zone, early-, mid- and late-pachytene.

Statistical evaluations were performed using the two-tailed t-Test using Microsoft Excel and the two-tailed Fisher's Exact test calculated as described [65].

## Supporting Information

Figure S1 Immunostaining detection of the SMC-5 and SMC-6 proteins in the *C. elegans* germline is specific. Micrographs of pachytene and diakinesis germ cells from the *smc-5* and *smc-6* mutants co-stained with antibodies to the cohesin SMC-3 protein (A–E) and the SMC-6 protein (A–C, E, F and I) or the SMC-5 protein (D, G and H). The *smc-5* and *smc-6* mutations specifically reduced the immunostaining for their cognate proteins (A–E, and H). The enrichment of the SMC-5 and SMC-6 proteins on diakinesis chromosomes appeared to be inter-dependent (F, G and I). The *smc-5(tm2868)* mutation may retain some biological function, therefore we cannot rule out the possibility that some residual SMC-5/6 proteins are still present, but are below the limit of detection by immunostaining. Scale bars = 5 µm.
(1.41 MB TIF)

Figure S2 The *smc-5* and *smc-6* mutants exhibited compromised germline functions. (A) Transgenerational sterility appeared in the *smc-5(ok2421)* and *smc-6(ok3294)* homozygous mutant strains but was absent in the wild-type and the *brc-1(tm1145)* mutant strains. Individual L4 hermaphrodites were isolated to establish independent colonies (n = 19 to 20 colonies per genotype). For the mutant strains, the L4 hermaphrodites used to establish the colonies were homozygous F1 mutants produced by heterozygous mutant parents. Each colony was then maintained for 12 generations, during which time five L4 hermaphrodites from each plate were transferred to a new plate every four to five days to allow the next generation of offspring to develop into L4 larvae. If a plate representing an independent colony has less than five viable L4 progeny, then the line is considered to be “sterile”. The line-graph presents the percentages of the starting independent colonies that are sterile at each generation. (B) The *smc-5* and *smc-6* mutants produced fewer fertilized eggs compared to the wild-type strain. Fertilized eggs produced by individual hermaphrodites were counted every six to 12 hours for four days starting from the late L4 larval stage. Ten or more hermaphrodites were analyzed per genotype, generation and RNAi treatment condition. The bar graph represents the average of the total eggs produced by an individual hermaphrodite per genotype/RNAi condition, and the error bars represent the SEMs. (C) A comparison of the average number of eggs produced per day between age-matched wild-type (n = 6) and the *smc-5(tm2868)* homozygous F1 mutant strain (n = 17). The color key shown in (C) also applies to (D and E). (D) Gonads from the wild-type and the *smc-5(tm2868)* mutant hermaphrodites at the specified ages were dissected, DAPI-DNA stained and visualized on a compound epifluorescence microscope for the presence or absence of sperm in the spermatheca. 12 to 21 hermaphrodites were examined for each genotype. (E) The *smc-5(tm2868)* mutant hermaphrodites were allowed to mate with males marked by a P*myo-2*::GFP reporter for 24 hours. Following the mating procedure, individual hermaphrodites were then transferred to separate plates and the number of larval offspring produced was counted. Mated hermaphrodites were identified based on the presence of male and GFP-positive larval offspring, and vice versa for unmated hermaphrodites. Hermaphrodites that did not produce any offspring were excluded from the analysis. The bar graph represents the average number of offspring produced by mated (n = 24) and unmated (n = 12) *smc-5(tm2868)* F1 mutant hermaphrodites, and the error bars represent the SEMs.
(0.51 MB TIF)

Figure S3 The *smc-5* and *smc-6* F1 mutants have smaller gonads with few germ cells in comparison to the wild-type. (A–D) Micrographs of dissected DAPI-stained gonads from age-matched wild-type and *smc-5* and *smc-6* mutants are shown at the same magnification. The white bracket indicates the approximate length of the gonad arm from the distal tip to the end of pachytene.
(0.41 MB TIF)

Figure S4 Aberrant RAD-51 focal staining is found in the pre-meiotic region in the *smc-5* and *smc-6* mutants. (A–E) Micrographs of DAPI and RAD-51 antibody staining in dissected gonads. The genotypes are indicated at the top of each set of micrographs. The white dashed lines mark the pre-meiotic regions. Scale bars = 5 µm. (F) The average numbers of RAD-51 foci per nucleus are presented in the bar graph. The error bars represent the SEMs.
(2.48 MB TIF)

Figure S5 ZHP-3 localization appeared normal in the *smc-5* and *smc-6* mutants. Micrographs of DAPI and ZHP-3 antibody stained germ cells at the late pachytene stage. The genotypes are indicated at the top of each set of micrographs. The average numbers of ZHP-3 foci per late pachytene germ cell (± SEM) are indicated for the wild-type, the *smc-5(ok2421)* and the *smc-6(ok3294)* mutants. Scale bars = 5 µm.
(0.78 MB TIF)

Figure S6 The *smc-5* and *smc-6* mutant oocytes exhibit chromosome dismorphology resembling defects seen in the *rad-51(lg8701)* mutant. (A) Micrographs of diakinesis chromosomes visualized by DAPI-DNA fluorescence in which the chromosomes failed to resolve properly in the *rad-51(lg8701)*, *smc-5(ok2421)* and *smc-6(ok3294)* mutants. (B) The bar graph represents the percentages of oocytes at the “−1” to “−3” positions of the gonad with less than 4 resolved DNA bodies (n = 30 oocytes per genotype). (C) The fragmentation defect of the *smc-5(ok2421)* and *smc-6(ok3294)* mutants were not enhanced by the cohesin *him-1(e879)* mutation. For each genotype, embryos were harvested and grown at the permissive temperature of 15-degree C for the *him-1(e879)* mutation until the worms had developed into late-stage L4 larvae. The worms were then shifted to the restrictive temperature of 25-degree C for 16 hours to disrupt cohesin function [1], before they were dissected and analyzed for the presence of DAPI-stained chromosome fragments. The difference in growth temperature had no obvious effects on the frequency of chromosome fragmentation in the *smc-5(ok2421)* and *smc-6(ok3294)* single mutants. More importantly, the *him-1(e879)* mutation did not enhance the fragmentation defect in the double mutants with either the *smc-5(ok2421)* and the *smc-6(ok3294)* mutant (Fisher's Exact Test, p values >0.8). The measurement counts and statistical comparisons are summarized in Table S5. [1] Chan RC, Chan A, Jeon M, Wu TF, Pasqualone D, et al. (2003) Chromosome cohesion is regulated by a clock gene paralogue TIM-1. Nature 423:1002–1009.
(0.29 MB TIF)

Table S1 Average germ corpses per gonad measured by acridine-orange staining. Acridine-orange staining was carried out by incubating the worms in a M9 solution containing 50 µg/mL of acridine-orange (Anaspec) for 3 hours followed by washes and destaining with additional M9 solution as described [2]. The RNAi inactivation of pro-apoptotic genes was performed by the feeding RNAi method [3], for which the worms were fed on the RNAi vector containing bacteria for two successive generations. [2] Gartner A, MacQueen AJ, Villeneuve AM (2004) Methods for analyzing checkpoint responses in *Caenorhabditis elegans*. Methods Mol Biol 280:257–274. [3] Kamath RS, Ahringer J (2003) Genome-wide RNAi screening in *Caenorhabditis elegans*. Methods 30:313–321.
(0.03 MB DOC)

Table S2 Average germ corpses per gonad measured by CED-1::GFP.
(0.03 MB DOC)

Table S3 Average number of RAD-51 foci per nucleus ± SEM in different regions of the germline from wild-type and mutant hermaphrodites. The sample size number (n) indicates the number of germ nuclei examined for each region per genotype.
(0.03 MB DOC)

Table S4 Average number of similar size DAPI-stained DNA figures in diakinesis oocytes.
(0.03 MB DOC)

Table S5 Proportion of oocytes with one or more chromosome fragments at diakinesis. Statistical comparisons were performed using the Fisher's Exact Test [4]. [4] Agresti A (1992) A survey of exact inference for contingency tables. Statistical Science 7:131–153.
(0.07 MB DOC)
