# Supplementary figures and images for: Structural Maintenance of Chromosomes (SMC) Proteins Promote Homolog-Independent Recombination Repair in Meiosis Crucial for Germ Cell Genomic Stability
Source: PLoS Genet. 2010 Jul 22;6(7):e1001028. doi: 10.1371/journal.pgen.1001028 (PMC2908675; doi:10.1371/journal.pgen.1001028)

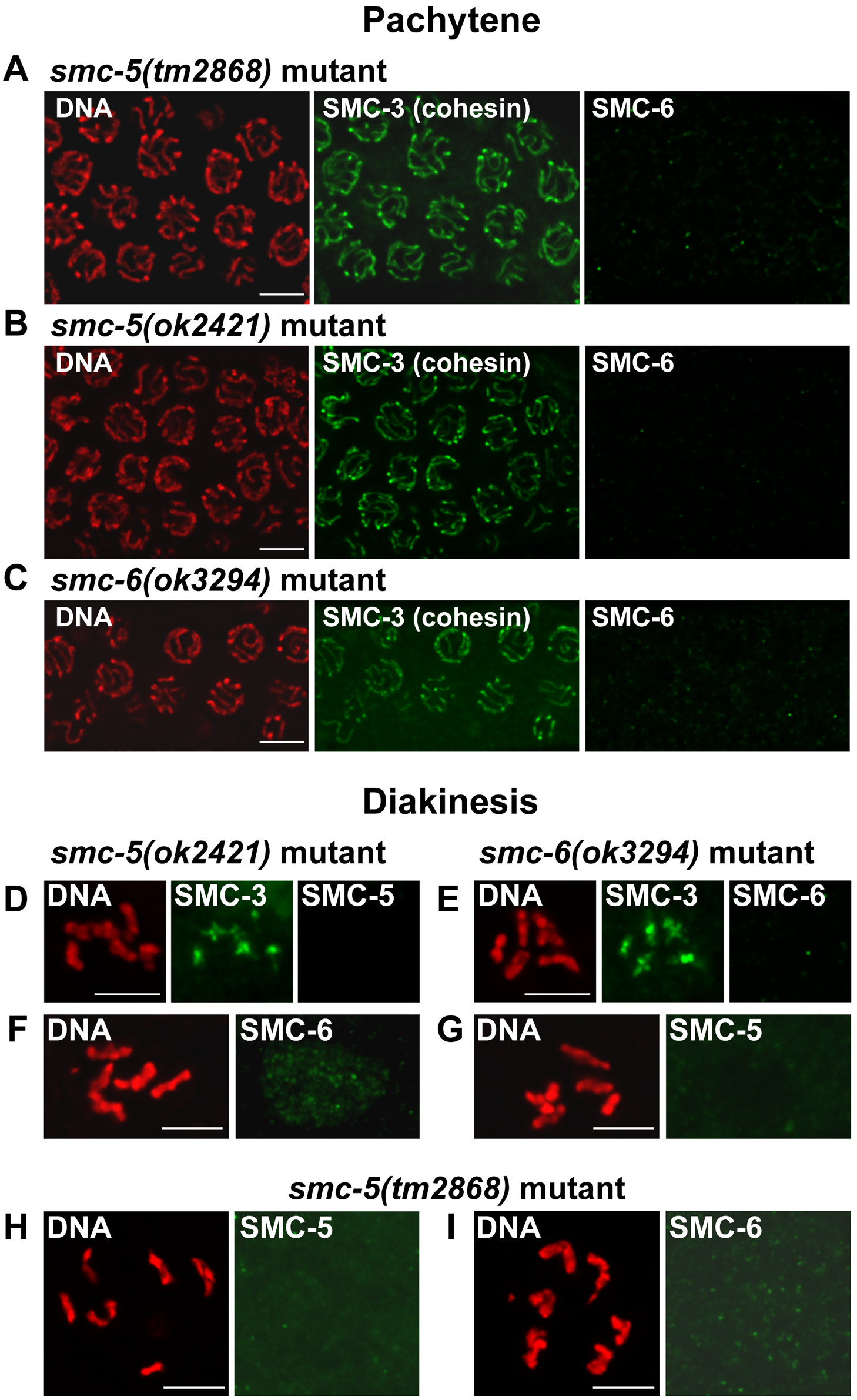

Supplement: Figure S1 — Immunostaining detection of the SMC-5 and SMC-6 proteins in the C. elegans germline is specific. Micrographs of pachytene and diakinesis germ cells from the smc-5 and smc-6 mutants co-stained with antibodies to the cohesin SMC-3 protein (A–E) and the SMC-6 protein (A–C, E, F and I) or the SMC-5 protein (D, G and H). The smc-5 and smc-6 mutations specifically reduced the immunostaining for their cognate proteins (A–E, and H). The enrichment of the SMC-5 and SMC-6 proteins on diakinesis chromosomes appeared to be inter-dependent (F, G and I). The smc-5(tm2868) mutation may retain some biological function, therefore we cannot rule out the possibility that some residual SMC-5/6 proteins are still present, but are below the limit of detection by immunostaining. Scale bars = 5 µm. (1.41 MB TIF) [file pgen.1001028.s001.tif]

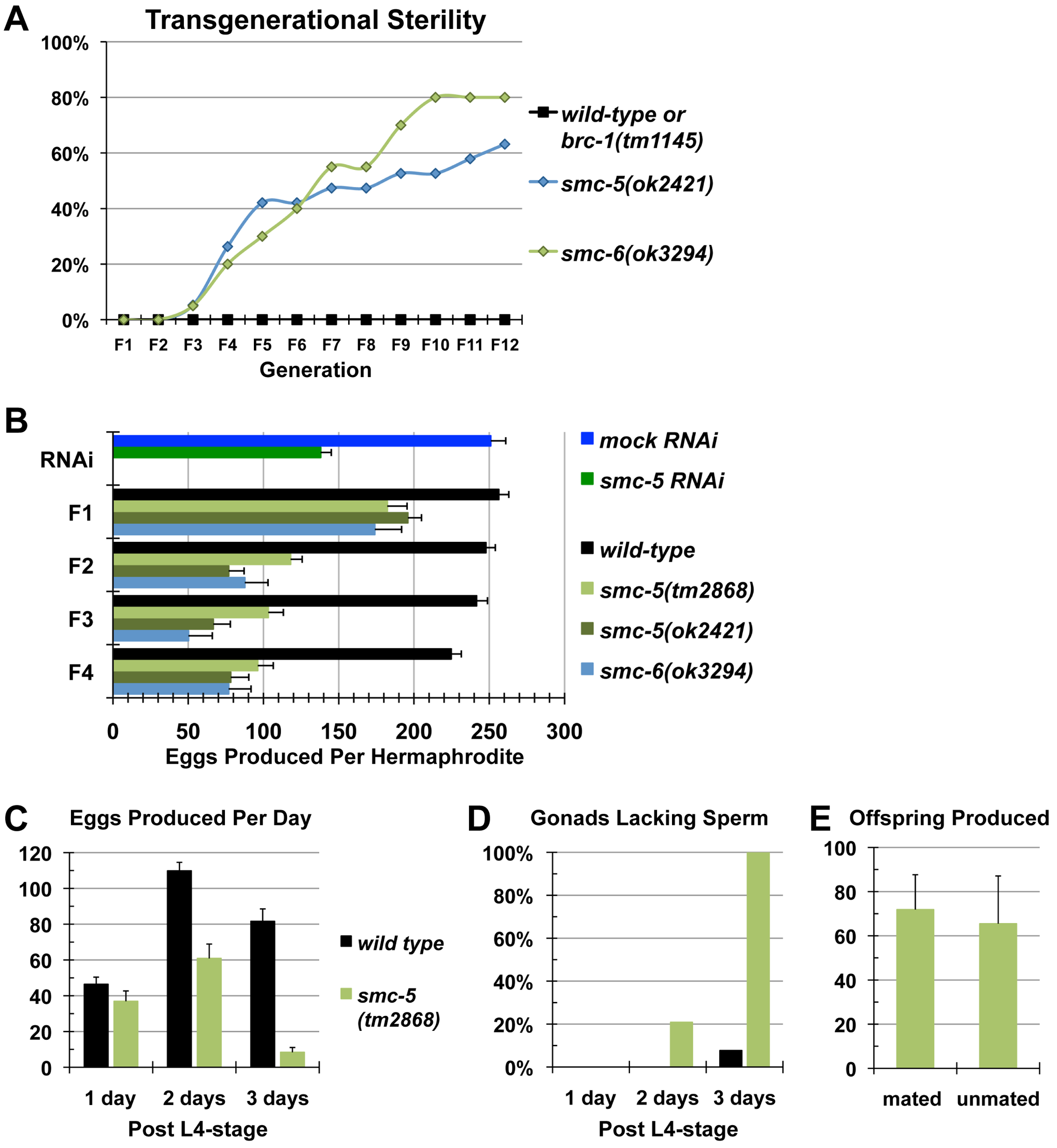

Supplement: Figure S2 — The smc-5 and smc-6 mutants exhibited compromised germline functions. (A) Transgenerational sterility appeared in the smc-5(ok2421) and smc-6(ok3294) homozygous mutant strains but was absent in the wild-type and the brc-1(tm1145) mutant strains. Individual L4 hermaphrodites were isolated to establish independent colonies (n = 19 to 20 colonies per genotype). For the mutant strains, the L4 hermaphrodites used to establish the colonies were homozygous F1 mutants produced by heterozygous mutant parents. Each colony was then maintained for 12 generations, during which time five L4 hermaphrodites from each plate were transferred to a new plate every four to five days to allow the next generation of offspring to develop into L4 larvae. If a plate representing an independent colony has less than five viable L4 progeny, then the line is considered to be “sterile”. The line-graph presents the percentages of the starting independent colonies that are sterile at each generation. (B) The smc-5 and smc-6 mutants produced fewer fertilized eggs compared to the wild-type strain. Fertilized eggs produced by individual hermaphrodites were counted every six to 12 hours for four days starting from the late L4 larval stage. Ten or more hermaphrodites were analyzed per genotype, generation and RNAi treatment condition. The bar graph represents the average of the total eggs produced by an individual hermaphrodite per genotype/RNAi condition, and the error bars represent the SEMs. (C) A comparison of the average number of eggs produced per day between age-matched wild-type (n = 6) and the smc-5(tm2868) homozygous F1 mutant strain (n = 17). The color key shown in (C) also applies to (D and E). (D) Gonads from the wild-type and the smc-5(tm2868) mutant hermaphrodites at the specified ages were dissected, DAPI-DNA stained and visualized on a compound epifluorescence microscope for the presence or absence of sperm in the spermatheca. 12 to 21 hermaphrodites were examined for each genotype. (E) [file pgen.1001028.s002.tif]

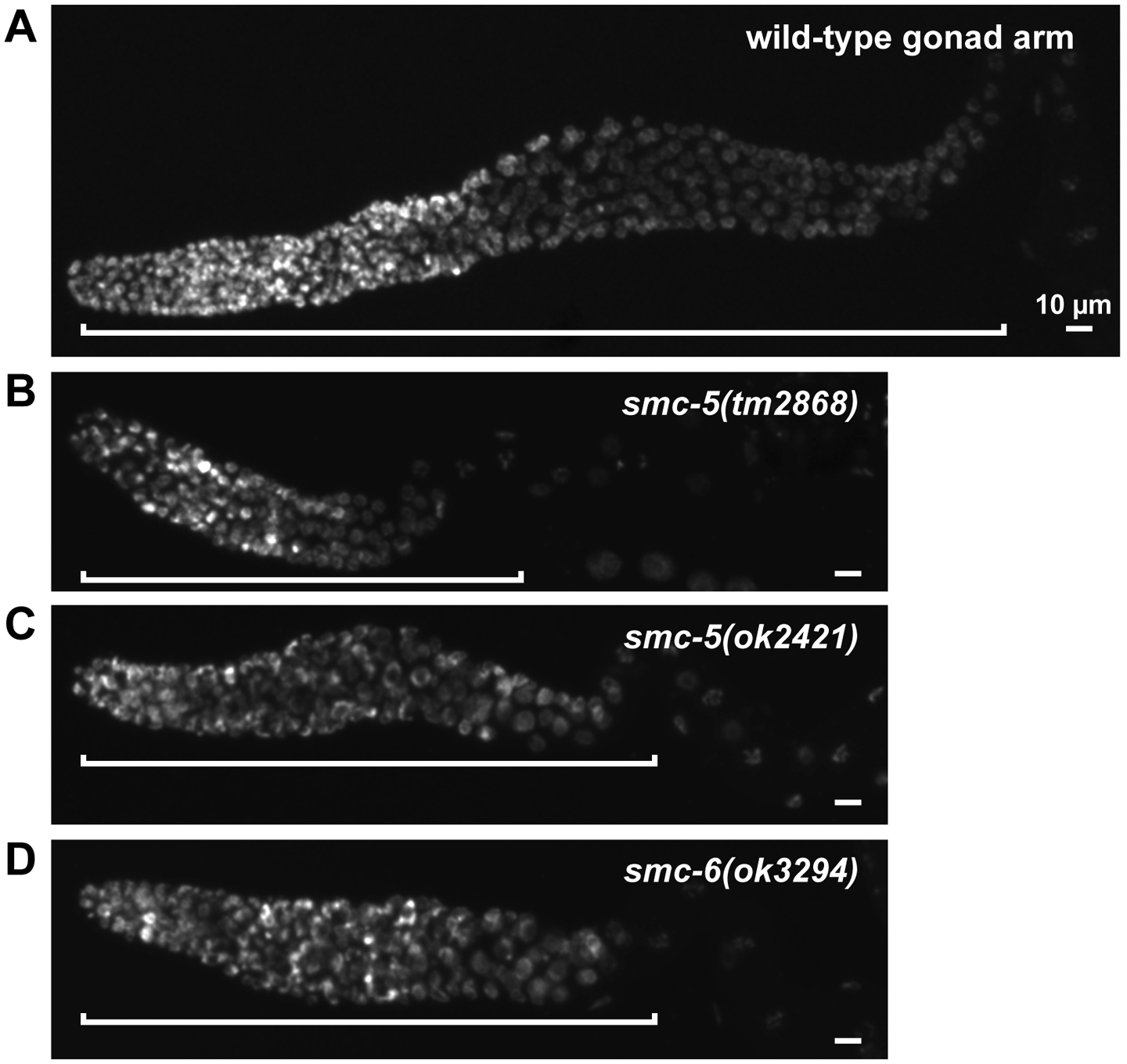

Supplement: Figure S3 — The smc-5 and smc-6 F1 mutants have smaller gonads with few germ cells in comparison to the wild-type. (A–D) Micrographs of dissected DAPI-stained gonads from age-matched wild-type and smc-5 and smc-6 mutants are shown at the same magnification. The white bracket indicates the approximate length of the gonad arm from the distal tip to the end of pachytene. (0.41 MB TIF) [file pgen.1001028.s003.tif]

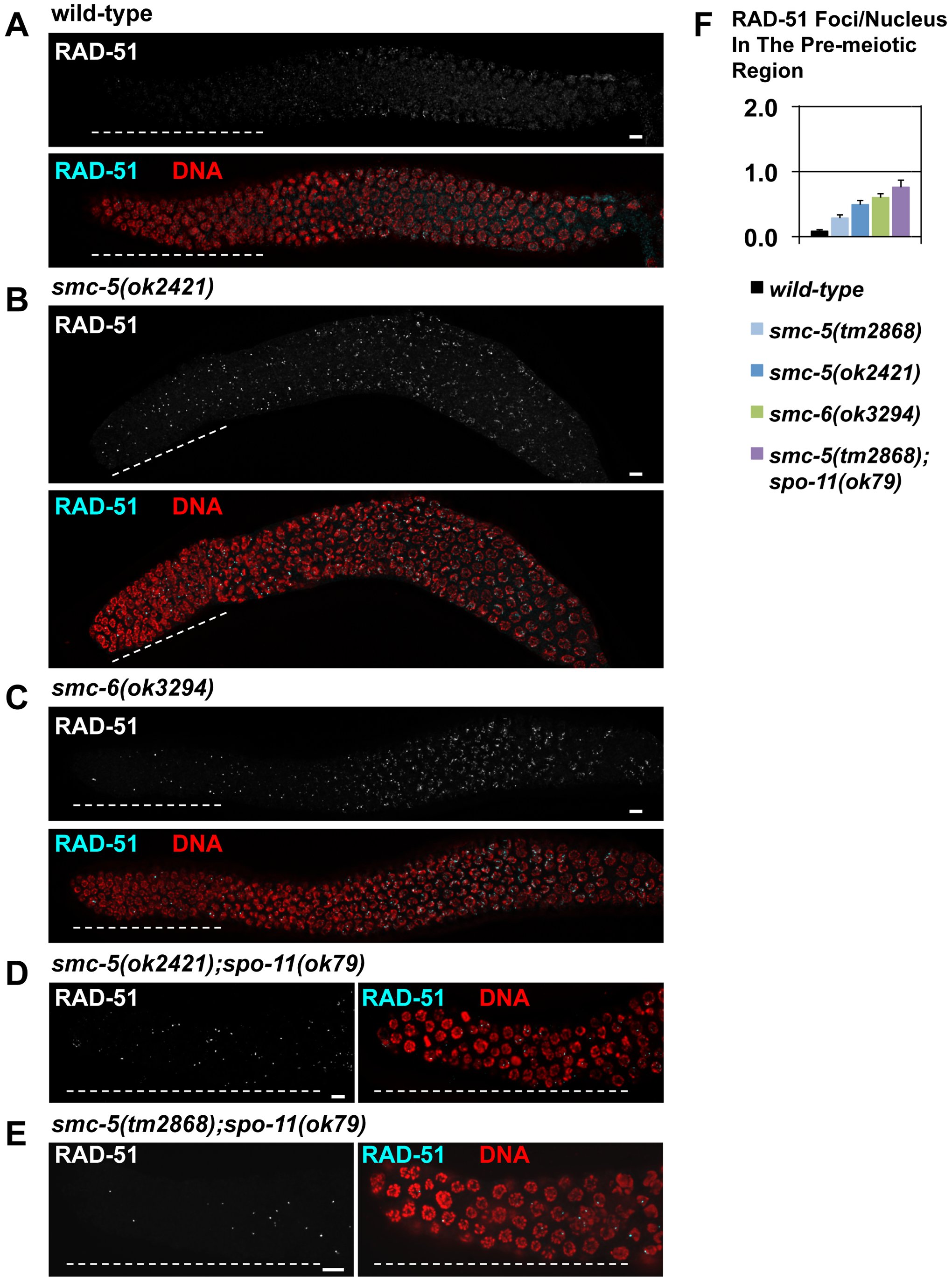

Supplement: Figure S4 — Aberrant RAD-51 focal staining is found in the pre-meiotic region in the smc-5 and smc-6 mutants. (A–E) Micrographs of DAPI and RAD-51 antibody staining in dissected gonads. The genotypes are indicated at the top of each set of micrographs. The white dashed lines mark the pre-meiotic regions. Scale bars = 5 µm. (F) The average numbers of RAD-51 foci per nucleus are presented in the bar graph. The error bars represent the SEMs. (2.48 MB TIF) [file pgen.1001028.s004.tif]

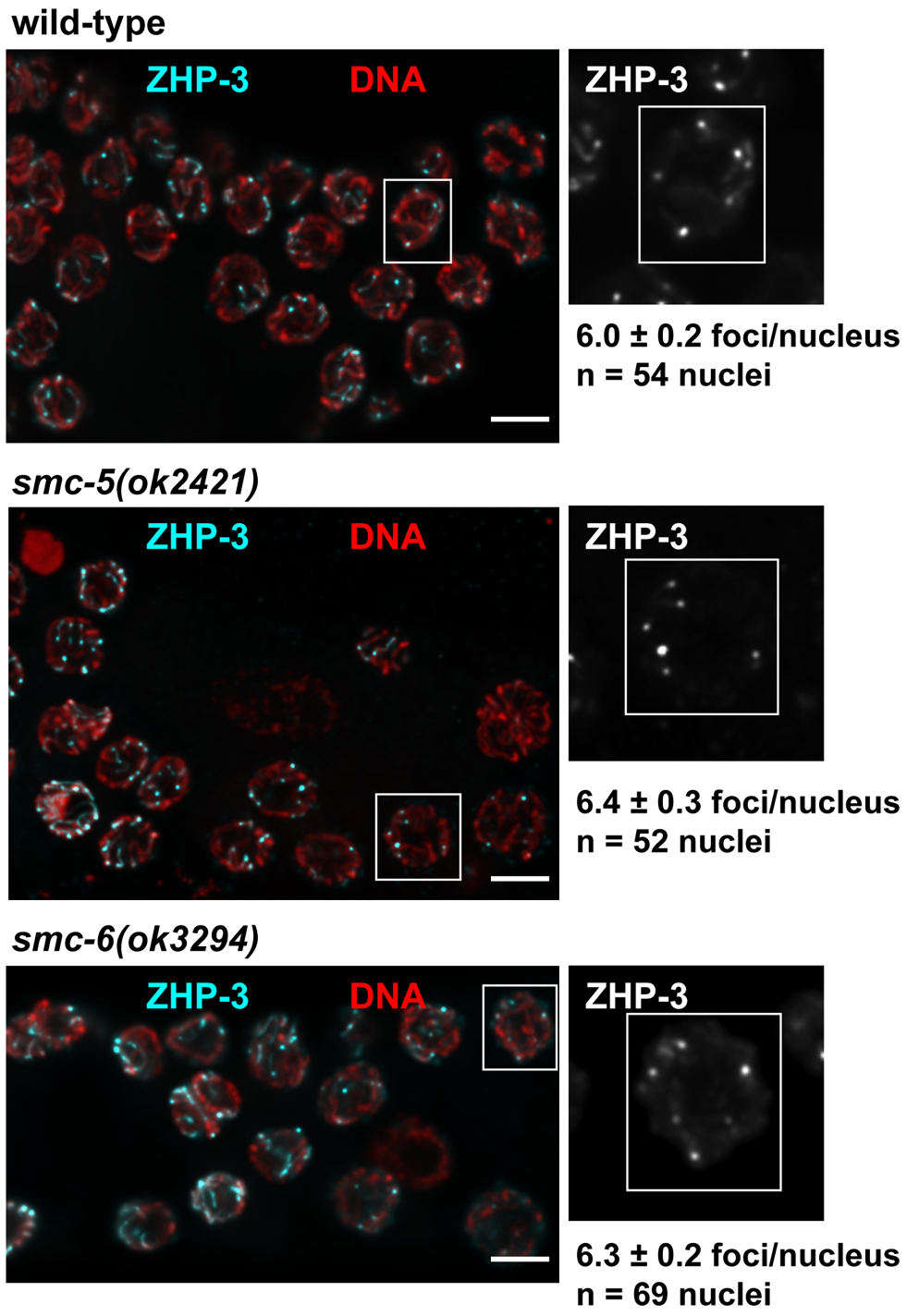

Supplement: Figure S5 — ZHP-3 localization appeared normal in the smc-5 and smc-6 mutants. Micrographs of DAPI and ZHP-3 antibody stained germ cells at the late pachytene stage. The genotypes are indicated at the top of each set of micrographs. The average numbers of ZHP-3 foci per late pachytene germ cell (± SEM) are indicated for the wild-type, the smc-5(ok2421) and the smc-6(ok3294) mutants. Scale bars = 5 µm. (0.78 MB TIF) [file pgen.1001028.s005.tif]

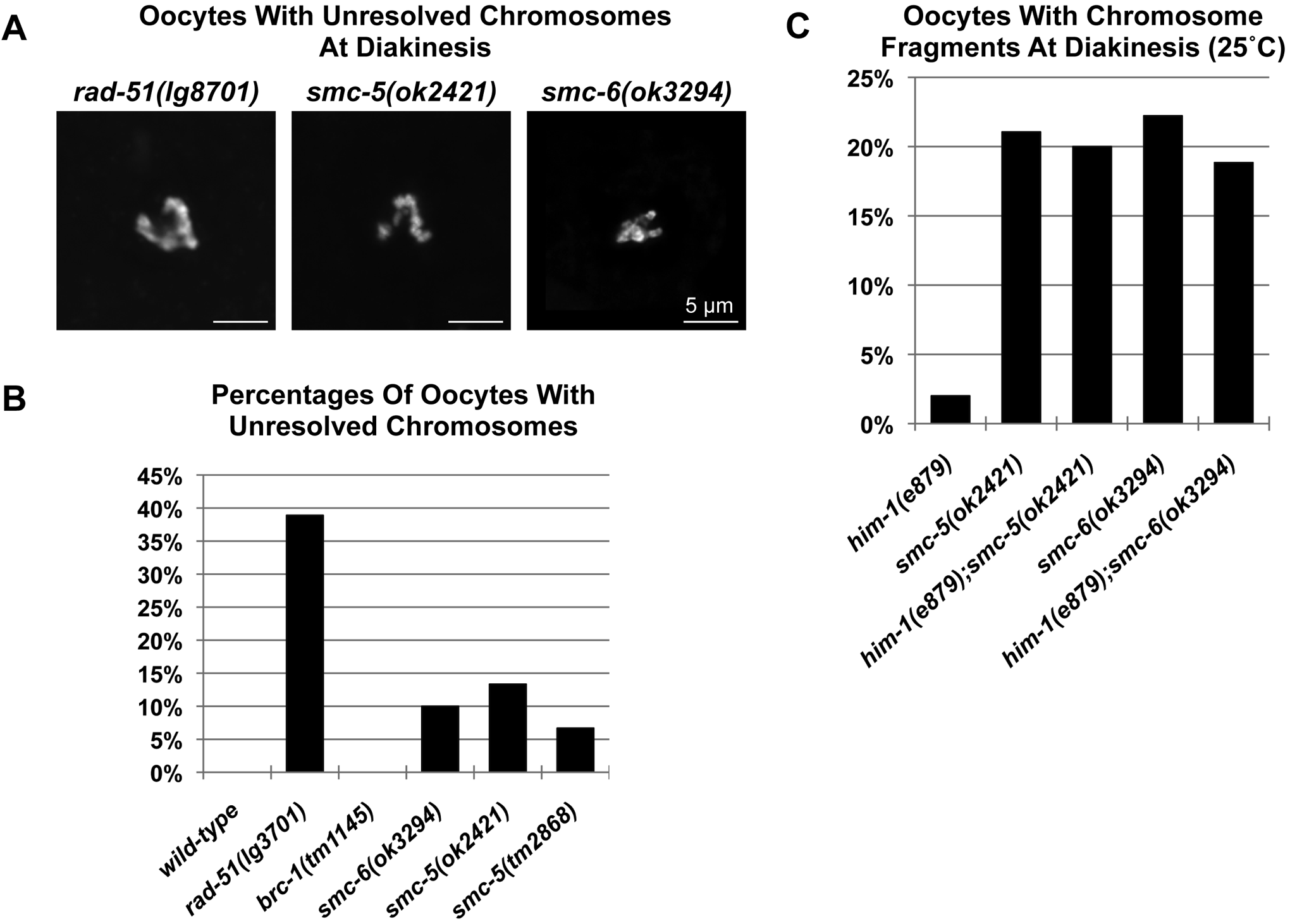

Supplement: Figure S6 — The smc-5 and smc-6 mutant oocytes exhibit chromosome dismorphology resembling defects seen in the rad-51(lg8701) mutant. (A) Micrographs of diakinesis chromosomes visualized by DAPI-DNA fluorescence in which the chromosomes failed to resolve properly in the rad-51(lg8701), smc-5(ok2421) and smc-6(ok3294) mutants. (B) The bar graph represents the percentages of oocytes at the “−1” to “−3” positions of the gonad with less than 4 resolved DNA bodies (n = 30 oocytes per genotype). (C) The fragmentation defect of the smc-5(ok2421) and smc-6(ok3294) mutants were not enhanced by the cohesin him-1(e879) mutation. For each genotype, embryos were harvested and grown at the permissive temperature of 15-degree C for the him-1(e879) mutation until the worms had developed into late-stage L4 larvae. The worms were then shifted to the restrictive temperature of 25-degree C for 16 hours to disrupt cohesin function [1], before they were dissected and analyzed for the presence of DAPI-stained chromosome fragments. The difference in growth temperature had no obvious effects on the frequency of chromosome fragmentation in the smc-5(ok2421) and smc-6(ok3294) single mutants. More importantly, the him-1(e879) mutation did not enhance the fragmentation defect in the double mutants with either the smc-5(ok2421) and the smc-6(ok3294) mutant (Fisher's Exact Test, p values >0.8). The measurement counts and statistical comparisons are summarized in Table S5. [1] Chan RC, Chan A, Jeon M, Wu TF, Pasqualone D, et al. (2003) Chromosome cohesion is regulated by a clock gene paralogue TIM-1. Nature 423:1002–1009. (0.29 MB TIF) [file pgen.1001028.s006.tif]
