# Supplementary material for: Structural Maintenance of Chromosomes (SMC) Proteins Promote Homolog-Independent Recombination Repair in Meiosis Crucial for Germ Cell Genomic Stability
Source: PLoS Genet. 2010 Jul 22;6(7):e1001028. doi: 10.1371/journal.pgen.1001028 (PMC2908675; doi:10.1371/journal.pgen.1001028)
Supplement: Table S1 — Average germ corpses per gonad measured by acridine-orange staining. Acridine-orange staining was carried out by incubating the worms in a M9 solution containing 50 µg/mL of acridine-orange (Anaspec) for 3 hours followed by washes and destaining with additional M9 solution as described [2]. The RNAi inactivation of pro-apoptotic genes was performed by the feeding RNAi method [3], for which the worms were fed on the RNAi vector containing bacteria for two successive generations. [2] Gartner A, MacQueen AJ, Villeneuve AM (2004) Methods for analyzing checkpoint responses in Caenorhabditis elegans. Methods Mol Biol 280:257–274. [3] Kamath RS, Ahringer J (2003) Genome-wide RNAi screening in Caenorhabditis elegans. Methods 30:313–321. (0.03 MB DOC) [file pgen.1001028.s007.doc]

| **Table S1. Germ Cell Corpses Detected By Acridine-Orange Staining** | | | | |  |
| --- | --- | --- | --- | --- | --- |
| **Genotype** | **wild-type** | ***smc-5 (tm2868)*** | ***smc-5 (ok2421)*** | ***smc-6 (ok3294)*** | ***smc-5(tm2868); ced-3(n717)*** |
| **Corpses per gonad ± SEM** | **2.0 ± 0.2** | **3.6 ± 0.2** | **3.3 ± 0.2** | **4.3 ± 0.3** | **0.4 ± 0.1** |
| **Gonad arms (n)** | **62** | **40** | **31** | **30** | **41** |
| **two-tailed t-Test (p value) compared to *wild-type*** |  | **< 0.001** | **< 0.001** | **< 0.001** |  |
| **compared to *smc-5(tm2868)*** |  |  |  |  | **< 0.001** |
