# Supplementary material for: Structural Maintenance of Chromosomes (SMC) Proteins Promote Homolog-Independent Recombination Repair in Meiosis Crucial for Germ Cell Genomic Stability
Source: PLoS Genet. 2010 Jul 22;6(7):e1001028. doi: 10.1371/journal.pgen.1001028 (PMC2908675; doi:10.1371/journal.pgen.1001028)
Supplement: Table S2 — Average germ corpses per gonad measured by CED-1::GFP. (0.03 MB DOC) [file pgen.1001028.s008.doc]

| **Table S2. Germ Cell Corpses Detected By Endocytosis (CED-1::GFP)** | | | | | | |
| --- | --- | --- | --- | --- | --- | --- |
| **Genotype** | **wild-type** | ***smc-5 (tm2868)*** | ***smc-5 (tm2868)*** | ***smc-5 (tm2868)*** | ***smc-5 (tm2868)*** |  |
| **RNAi** | **vector** | **vector** | ***ced-3*** | ***ced-4*** | ***egl-1*** |  |
| **Corpses per gonad ± SEM** | **7.1 ± 0.4** | **15.5 ± 0.9** | **7.2 ± 1.3** | **11.1 ± 1.0** | **11.8 ± 1.0** |  |
| **Gonad arms (n)** | **40** | **37** | **39** | **36** | **29** |  |
| **two-tailed t-Test (p value) compared to wild-type** |  | **< 0.001** |  |  |  |  |
| **compared to *smc-5* + vector RNAi** |  |  | **< 0.001** | **0.001** | **0.007** |  |
