# Supplementary material for: Structural Maintenance of Chromosomes (SMC) Proteins Promote Homolog-Independent Recombination Repair in Meiosis Crucial for Germ Cell Genomic Stability
Source: PLoS Genet. 2010 Jul 22;6(7):e1001028. doi: 10.1371/journal.pgen.1001028 (PMC2908675; doi:10.1371/journal.pgen.1001028)
Supplement: Table S3 — Average number of RAD-51 foci per nucleus ± SEM in different regions of the germline from wild-type and mutant hermaphrodites. The sample size number (n) indicates the number of germ nuclei examined for each region per genotype. (0.03 MB DOC) [file pgen.1001028.s009.doc]

| **Table S3. RAD-51 Foci Per Germ Nucleus** | | | | |  | |  | |
| --- | --- | --- | --- | --- | --- | --- | --- | --- |
| **Genotype** | **pre-meiotic** | **transition zone** | **early pachytene** | **mid pachytene** | | **late pachytene** | |  |
| **wild-type** | **0.1 ± 0.0 (n = 134)** | **0.6 ± 0.1 (n = 107)** | **3.0 ± 0.2 (n = 76)** | **1.5 ± 0.2 (n = 63)** | | **0.6 ± 0.1 (n = 51)** | |  |
| ***smc-5(tm2868)*** | **0.3 ± 0.0 (n = 123)** | **1.0 ± 0.1 (n = 104)** | **3.0 ± 0.2 (n = 114)** | **3.1 ± 0.3 (n = 80)** | | **1.4 ± 0.2 (n = 72)** | |  |
| ***smc-5(ok2421)*** | **0.5 ± 0.1 (n = 118)** | **1.1 ± 0.1 (n = 92)** | **3.9 ± 0.2 (n = 96)** | **5.1 ± 0.4 (n = 67)** | | **3.7 ± 0.5 (n = 58)** | |  |
| ***smc-6(ok3294)*** | **0.6 ± 0.1 (n = 175)** | **1.1 ± 0.1 (n = 112)** | **4.4 ± 0.2 (n = 76)** | **4.9 ± 0.2 (n = 70)** | | **3.9 ± 0.3 (n = 85)** | |  |
| ***smc-5(tm2868); spo-11(ok79)*** | **0.8 ± 0.1 (n = 83)** | **0.5 ± 0.1 (n = 119)** | **0.8 ± 0.1 (n = 99)** | **0.8 ± 0.1 (n = 69)** | | **0.7 ± 0.1 (n = 57)** | |  |
