# Supplementary material for: Structural Maintenance of Chromosomes (SMC) Proteins Promote Homolog-Independent Recombination Repair in Meiosis Crucial for Germ Cell Genomic Stability
Source: PLoS Genet. 2010 Jul 22;6(7):e1001028. doi: 10.1371/journal.pgen.1001028 (PMC2908675; doi:10.1371/journal.pgen.1001028)
Supplement: Table S4 — Average number of similar size DAPI-stained DNA figures in diakinesis oocytes. (0.03 MB DOC) [file pgen.1001028.s010.doc]

| **Table S4** |  |  |  |  |
| --- | --- | --- | --- | --- |
| **Genotype** | **wild-type** | ***smc-5 (tm2868)*** | ***smc-5 (ok2421)*** | ***smc-6 (ok3294)*** |
| **Linked Homologs Per Oocyte ± SD** | **5.9 ± 0.2** | **5.9 ± 0.3** | **6.1 ± 0.5** | **5.9 ± 0.4** |
| **oocytes (n)** | **94** | **59** | **53** | **53** |
