# Supplementary material for: Structural Maintenance of Chromosomes (SMC) Proteins Promote Homolog-Independent Recombination Repair in Meiosis Crucial for Germ Cell Genomic Stability
Source: PLoS Genet. 2010 Jul 22;6(7):e1001028. doi: 10.1371/journal.pgen.1001028 (PMC2908675; doi:10.1371/journal.pgen.1001028)
Supplement: Table S5 — Proportion of oocytes with one or more chromosome fragments at diakinesis. Statistical comparisons were performed using the Fisher's Exact Test [4]. [4] Agresti A (1992) A survey of exact inference for contingency tables. Statistical Science 7:131–153. (0.07 MB DOC) [file pgen.1001028.s011.doc]

| **Table S5** |  |  |  |  |  |  |
| --- | --- | --- | --- | --- | --- | --- |
| **Genotype** | **Oocytes** | | **Fisher's Exact Test, p-values** | | | |
|  | **Containing Fragment(s)** | **Total (n)** | **wild-type *(N2)*** | ***smc-6 (ok3294)*** | ***smc-5 (ok2421)*** | ***smc-5 (tm2868)*** |
| **wild-type *(N2)*** | 0.0% | 94 |  |  |  |  |
| ***smc-6(ok3294)*** | 16.0% | 75 | < 0.01 |  |  |  |
| ***smc-5(ok2421)*** | 20.5% | 78 | < 0.01 |  |  |  |
| ***smc-5(ok2421);spo-11(ok79)*** | 2.4% | 41 |  |  | < 0.01 |  |
| ***smc-5(tm2868)*** | 1.5% | 68 |  |  |  |  |
| ***rad-51(lg8701)*** | 15.2% | 33 |  |  |  |  |
| ***spo-11(ok79)*** | 0.0% | 61 |  |  |  |  |
| ***him-3(gk149)*** | 1.5% | 65 |  |  |  |  |
| ***smc-5(tm2868);him-3(gk149)*** | 20.0% | 95 |  |  |  | < 0.01 |
| ***smc-5(ok2421);him-3(gk149)*** | 30.2% | 53 |  |  | 0.22 |  |
| ***smc-6(ok3294);him-3(gk149)*** | 33.0% | 103 |  | 0.01 |  |  |
| ***syp-2(RNAi)*** | 1.4% | 69 |  |  |  |  |
| ***smc-5(ok2421) + vector RNAi*** | 21.3% | 80 |  |  |  |  |
| ***smc-5(ok2421);syp-2(RNAi)*** | 30.5% | 82 |  |  | 0.21 |  |
| ***smc-6(ok3294) + vector RNAi*** | 21.0% | 62 |  |  |  |  |
| ***smc-6(ok3294);syp-2(RNAi)*** | 38.7% | 62 |  | 0.05 |  |  |
| ***brc-1(tm1145)*** | 4.8% | 84 |  |  |  |  |
| ***smc-5(ok2421);brc-1(tm1145)*** | 16.4% | 55 |  |  | 0.65 |  |
| ***him-1(e879)*** | 2.0% | 50 |  |  |  |  |
| ***smc-5(ok2421)*** | 21.1% | 38 |  |  |  |  |
| ***him-1(e879);smc-5(ok2421)*** | 20.0% | 70 |  |  | 1.00 |  |
| ***smc-6(ok3294)*** | 22.2% | 27 |  |  |  |  |
| ***him-1(e879);smc-6(ok3294)*** | 18.8% | 69 |  | 0.78 |  |  |
